# Functions of Ceramide Synthase Paralogs YPR114w and YJR116w of *Saccharomyces cerevisiae*

**DOI:** 10.1371/journal.pone.0145831

**Published:** 2016-01-11

**Authors:** Shamroop K. Mallela, Reinaldo Almeida, Christer S. Ejsing, Andreas Conzelmann

**Affiliations:** 1 Division of Biochemistry, Department of Biology, University of Fribourg, Chemin du Musée 10, Fribourg, CH-1700, Switzerland; 2 Department of Biochemistry and Molecular Biology, University of Southern Denmark, Campusvej 55, DK-5230, Odense M, Denmark; Medical University of South Carolina, UNITED STATES

## Abstract

Ceramide is synthesized in yeast by two redundant acyl-CoA dependent synthases, Lag1 and Lac1. In *lag1∆ lac1∆* cells, free fatty acids and sphingoid bases are elevated, and ceramides are produced through the redundant alkaline ceramidases Ypc1 and Ydc1, working backwards. Even with all four of these genes deleted, cells are surviving and continue to contain small amounts of complex sphingolipids. Here we show that these residual sphingolipids are not synthesized by YPR114w or YJR116w, proteins of unknown function showing a high degree of homology to Lag1 and Lac1. Indeed, the hextuple *lag1∆ lac1∆ ypc1∆ ydc1∆ ypr114w∆ yjr116w∆* mutant still contains ceramides and complex sphingolipids. *Yjr116w∆* exhibit an oxygen-dependent hypersensitivity to Cu^2+^ due to an increased mitochondrial production of reactive oxygen species (ROS) and a mitochondrially orchestrated programmed cell death in presence of copper, but also a general copper hypersensitivity that cannot be counteracted by the antioxidant N-acetyl-cysteine (NAC). Myriocin efficiently represses the synthesis of sphingoid bases of *ypr114w∆*, but not its growth. Both *yjr116w∆* and *ypr114w∆* have fragmented vacuoles and produce less ROS than wild type, before and after diauxic shift. *Ypr114w∆/ypr114w∆* have an increased chronological life span. Thus, Yjr116w and Ypr114w are related, but not functionally redundant.

## Introduction

**Fig 1:**
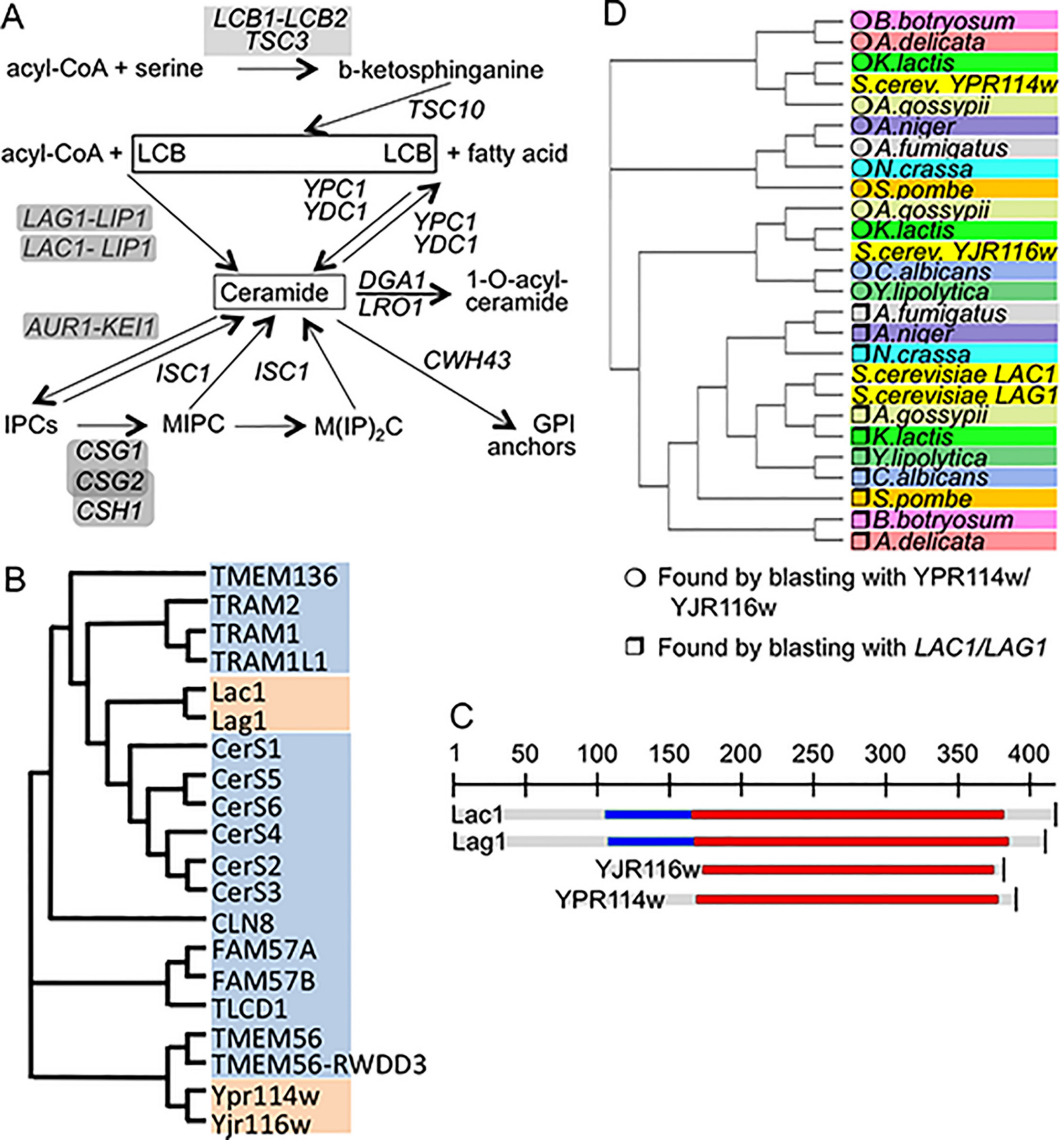
Genes in focus. A. Pathways of sphingolipid biosynthesis and degradation in yeast. Gene names are in italics, for enzymes requiring complex formation, genes are underlain in grey. B. Cladogram of ceramide synthases and their TLC domain containing paralogs of yeast (orange) and humans (blue). C. Alignment of *LAG1*, *LAC1*, YJR116w and YPR114w. The TLC domain is shown in red, the TRAM domain in blue. D. Homologues of *LAG1*, *LAC1*, YJR116w and YPR114w in intensively studied fungal species were found using PSI-Blasts (http://blast.ncbi.nlm.nih.gov/) as described in Table A in S1 File. The cladogram was generated by clustal W (http://www.ebi.ac.uk/Tools/msa/clustalw2/).

The yeast sphingolipids are generated by the pathways shown in Fig 1A and are essential structural components of cell membranes.

They also act as messengers regulating the proliferation, survival, aging and death of cells [1,2]. The long chain bases (LCBs) dihydrosphingosine (DHS) and its 4-hydroxy derivative, phytosphingosine (PHS) are made and attached to fatty acids to form ceramides in the ER. The biosynthesis of inositolphosphorylceramides (IPCs) and more complex sphingolipids is believed to occur in the Golgi. Thus, ceramide is an intermediate in the formation of complex sphingolipids. The acyl-CoA dependent biosynthesis of ceramide is operated by Lag1 and Lac1 (Fig 1A), two highly homologous and functionally redundant ER proteins, which are only active when forming a complex with Lip1 [3–5]. Concomitant deletion of *LAG1* and *LAC1* causes a significant growth defect in the W303 genetic background and the same double deletion is lethal in the YPK9 background [6,7]. When *LAG1* and *LAC1* are deleted, ceramide levels drop and the LCBs reach very high concentrations [3]. In these conditions it becomes energetically possible that ceramides are made by the ER based alkaline ceramidases Ypc1p and Ydc1p through a reverse reaction condensing free fatty acids with LCBs [8–13]. Several reports indicated however that W303 *lag1∆ lac1∆ ypc1∆ ydc1∆* (W303.4∆) cells are not only viable, but still are able to synthesize low amounts of sphingolipids and to incorporate close to normal amounts of ceramides into GPI anchors [10,14]. These data suggest that yeast possess yet alternative ways of making ceramides.

The yeast genome contains two open reading frames (ORFs), which are distantly homologous to *LAG1* and *LAC1*, YPR114w and YJR116w. Pairwise comparisons of YPR114w and YJR116w with *LAG1* or *LAC1* show only 11–17% of identities, even when only the characteristic, 200 amino acids long, conserved *TRAM1-LAG1-CLN8* (TLC) domain is considered, but the profile-profile comparison tool HHpred (http://toolkit.tuebingen.mpg.de/hhpred) predicts *LAG1* to be homologous with YJR116w and YPR114w with P values of 2.5 x 10^−33^ and 5.3 x 10^−21^, respectively (Fig 1B and 1C). Similar to *LAG1* and *LAC1*, YJR116w and YPR114w belong to the pfam PF03798 and the smart00724 gene families, the former comprising 1332 sequences from 246 species, all containing the TLC domain. However, unlike *LAG1* and *LAC1*, YPR114w and YJR116w do not contain a TRAM domain upstream of the TLC domain (Fig 1C), a domain which also is absent from all human ceramide synthases (CerS1 to CerS6, Fig 1B). YJR116w and YPR114w also retain only the first His of a conserved His-His motif present in all ascertained ceramide synthases [4]. Interestingly, only the mutation of this first His residues has previously been shown to strongly reduce/abolish ceramide synthase activities, whereas mutation to Asp of only the second His left some residual activity [15,16]. This suggests that the one His present in YPR114w and YJR116w may be sufficient for ceramide synthase activity. TLC-domain proteins closer to YPR114w and/or YJR116w than to *LAG1* and/or *LAC1* are conserved in many fungal species and are duplicated also in *K*.*lactis* and *A*.*gossypii* (Fig 1D).

Here we investigated if YPR114w and YJR116w are involved in the biosynthesis of ceramides and sphingolipids.

## Materials and Methods

### Chemicals and materials

Aureobasidin A was obtained from Takara Shuzo Co, tunicamycin from Sigma Aldrich, FM4-64 from Molecular probes (T-13320), dihydroethidium (DHE) from Marker gene technologies. Calcoflour white (CFW), myriocin, quinacrine, and N-acetyl-L-cysteine (NAC) from Sigma-Aldrich, [^3^H]*myo*-inositol from ANAWA Trading SA. Anti-Kar2 and anti-Gas1 antibodies were the kind gifts of Drs. M. Rose and F.Reggiori, anti-CPY antibodies (A-6428) were from Molecular Probes. Secondary antibodies were anti-rabbit IgG peroxidase conjugate (Sigma A6154) and anti-mouse IgG peroxidase conjugate (Sigma A4416). PVDF membranes were obtained from Millipore, Cat.No IPVH00010.

### Yeast strains, plasmids, growth media

Strains and plasmids used in this study are listed in the Tables B and C in S1 File. Cells were grown at 30°C on YP (1% yeast extract, 2% peptone) or on synthetic complete (SC) medium (Yeast nitrogen base (YNB) from United States Biological) plus amino acids, containing either 2% glucose (D) or galactose (Gal). 4∆, 6∆ and control cells were grown on Lester medium (LM)[14] containing 4% glucose or 4% galactose with 50 mg/liter inositol and 50 mM sodium succinate, pH 6.0. All experiments were done with strains having the BY4741/ 2 background growing exponentially at 30°C unless indicated otherwise.

### Analysis of lipids by mass spectrometry (MS)

Exponentially growing cells were extracted and analyzed in negative and positive ion mode by direct infusion [17,18] or normal-phase liquid chromatography (NPLC)[19] using an LTQ Orbitrap XL mass spectrometer equipped with a Triversa NanoMate ion source (Advion Biosciences). Data were expressed either as intensity profiling (lipid analyte intensity normalized to the sum of intensities of all monitored lipid analytes) or as mol% (based on use of internal lipid standards)[18,20].

To measure LCBs, cells were sedimented, resuspended in 1 ml of 150 mM NH_4_HCO_3_ (pH 8.0) and cells were lysed using glass beads, at 4°C. Lysates were transferred into 30 ml Pyrex tubes and 2 ml of 150 mM NH_4_HCO_3_ (pH 8.0) and 5 ml of 2:1 (chloroform:methanol) were added. At this step C17-DHS was added as an internal standard. The samples were vortexed for 30 s every 20 min during 2 h at 4°C. Tubes were centrifuged at 1000 x g and organic phases were collected and dried. Lipids were resuspended in 2:1 (chloroform:methanol) and analyzed by ESI-MS by direct infusion into a Bruker Daltonics Esquire HCT, at 280°C in the positive ion mode. We screened for DHS18 = LCB18:0;2 (*m/z* 302.3), PHS18 = LCB18:0;3 (*m/z* 318.3), DHS20 = LCB20:0;2 (*m/z* 330.3), and PHS20 = LCB20:0;3 (*m/z* 346.3) and the intensity of the internal standard was used to calculate the molar amounts of each LCB.

### Sensitivity to drugs and metal ions

To assess sensitivity of yeast cultures to different drugs and metal ions, cells were grown to exponential phase (OD_600_ = 0.8) and 10 fold serial dilutions were plated on media having either drugs or metal ions at indicated concentrations. For growth measurements in liquid culture using Bioscreen C, cells were grown in YPD till exponential phase (OD_600_ = 0.8) and were used to inoculate fresh YPD medium with or without drugs to an initial OD at 600 nm of 0.2. Cultures were then transferred into sterile 96 well plates and growth curves were obtained at 30°C under intermittent shaking.

### Protein extraction and western blotting

Proteins were extracted from cells as described [21]. The extracted protein samples were separated by 10% SDS/PAGE gel and transferred onto a PVDF membrane.

### Microsocopy

To visualize vacuoles, log phase cells were collected and incubated with 50 μM FM4-64 in YPD for 30 min at 30°C, centrifuged, resuspended in YPD and incubated further for 120 min, washed twice with distilled water. Cells were viewed under an Olympus BX54 microscope equipped with a piezo-positioner using a FM4-64 filter. Dihydroethidium (DHE) was used to detect superoxide anions (O_2_^-^). For this, exponentially growing cells were incubated for 15 min with 10 μg ml^-1^ DHE in YPD at 30°C, washed twice with distilled water and viewed as above using the RFP filter as described [22].

### Chronological life span (CLS) analysis

Chronological life span of yeast cultures were measured exactly as previously described [13]. Briefly, cells were grown in SC to stationary phase for 3 days, washed and resuspended in H_2_O and further incubated at 30°C on a rotating wheel. Cells were washed with sterile water every 3 days and resuspended in fresh sterile water in order to prevent gasping (feeding on remains of deceased neighbors). Their viability was determined at different time intervals by plating on YPD plates and counting colony-forming units (CFU).

All other methods are described in Materials and Methods of the S1 File.

## Results

### The synthesis of IPC and MIPCs continues in the absence of known ceramide synthases

**Fig 2:**
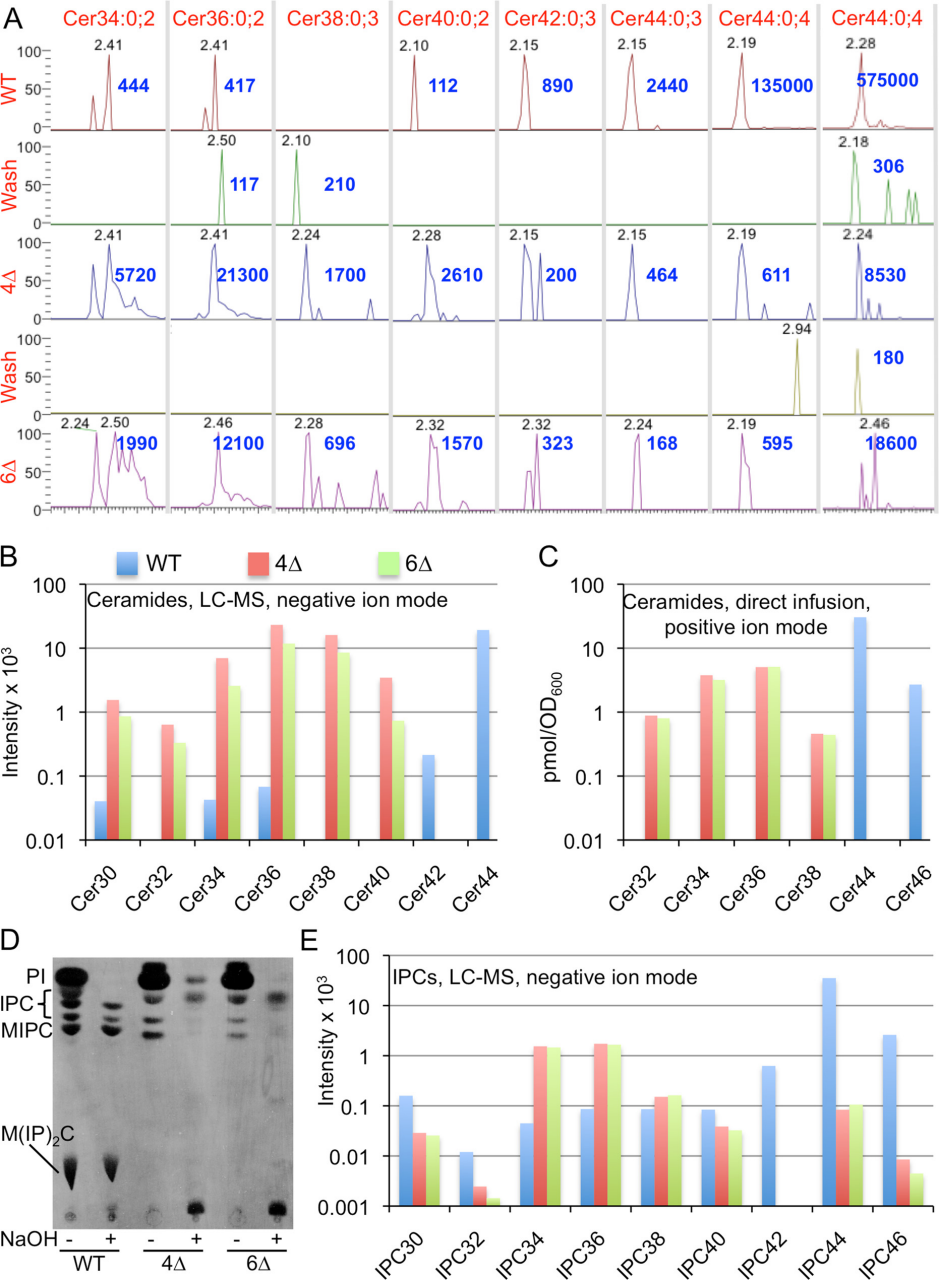
Analysis of ceramides and IPCs in WT, 4∆ and 6∆ by LC-MS(A, B and E) or by direct infusion MS (C). A. Extracted ion chromatograms (XIC) in negative ion mode of LC-MS analysis in time window where ceramides elute. (Cer44:0;4 is also shown in positive ion mode in the last column to the right). Elution times of peak signals for each ceramide species in minutes are in grey, apexes were set to 100% and the intensity at the apex indicated in blue. Only major species are shown. B. For experiment of panel A, the average intensity across the width of the entire LC peak for each ceramide species was determined and these averages for the various ceramides having a given number of C atoms were summed up and plotted. C. In an independent experiment lipid extracts were analyzed by direct infusion-FTMS and after addition of Cer35:1;3 as an internal standard. D. WT, 4∆ and 6∆ cells were labeled with [^3^H]inositol for 2 h and lipid extracts before and after deacylation were analyzed by TLC and autoradiography. E. In the experiment of panels A and B, IPCs were calculated and plotted as was done for ceramides in panel B.

Deletion of YPR114w or YJR116w or both did not significantly alter the lipid and sphingolipid profile of cells as judged by direct infusion mass spectrometry (MS)(not shown). To find out if Ypr114w and Yjr116w can act as ceramide synthases and are responsible for the persistence of complex sphingolipids in W303.4∆ cells [10,14], we deleted these genes in W303.4∆ *trp5*::*SLC1-1* (= 4∆) cells, thus generating the 4∆.W303 *trp5*::*SLC1-1 ypr114w∆ yjr116w∆* (= 6∆) strain (Table B in S1 File). The 6∆ strain grew as well as the parental 4∆ strain. NPLC-nanoESI-FTMS mass spectrometry (MS) analysis in positive and negative ion mode (LC-MS) showed that the main ceramide species in wild type (WT) cells is Cer44:0;4, a ceramide of 44 C atoms, 0 double bonds and 4 hydroxyl groups (Fig 2A).

This species corresponds to the main ceramide of yeast, made of a C18-PHS and a mono-hydroxylated C26 fatty acid. Small amounts of Cer44:0;4 are also detectable in both 4∆ and 6∆ cells (from 0.44 to 3.2% of WT levels, depending on ion mode)(Fig 2A). 4∆ and 6∆ cells also contained significant amounts of Cer34, Cer36, Cer38 and Cer40 (Fig 2A and 2B), indicating that the residual ceramide synthesis of 4∆ and 6∆ cells also uses fatty acids with 16 to 22 C atoms. These species are much less abundant in WT, most likely because Lag1 and Lac1 only use C24- and C26-CoA and use up the majority of LCBs. An independent experiment using direct infusion MS run with an internal ceramide standard, confirmed the observations of LC-MS analysis (Fig 2C).

Ceramides are also integrated into GPI anchors by GPI anchor lipid remodeling and this process is barely affected in 4∆ cells [14]. It therefore was hypothesized that ceramides for GPI remodeling may be made by enzymes different from Lag1, Lac1, Ypc1 and Ydc1. Data in S1 File (Fig A, panel A in S1 File) however show that the ceramide remodeling of GPI anchors occurs normally in *ypr114w∆*, *yjr116w∆* and yy∆∆ cells. Moreover, metabolic labeling with [^3^H]inositol showed that 6∆ cells still contain the same abnormal mild base resistant inositides (IPCs) as 4∆ cells (Fig 2D). Fig 2E and S1 File (Fig B, panel A in S1 File) show quantifications of IPCs detectable by MS and demonstrate that 4∆ as well as 6∆ cells both contain significantly elevated amounts of IPC34 and IPC36 and possibly also small amounts of IPC44s. IPC44s and MIPC44s in 4∆ and 6∆ were clearly identified in deacylated lipid extracts analyzed by direct infusion-FTMS (Fig B, panels B and C in S1 File). IPC (*m/z* 952.69) and of MIPC (*m/z* 1114.74) signals of 4∆ strain yielded the characteristic inositol-phosphate mannosyl-inositol-phosphate fragments (not shown). Moreover, direct infusion mass spectrometry of lipid extracts of 5∆ mutants, i.e. of W303.4∆ *trp5*::*SLC1-1 ypr114w∆* and W303.4∆ *trp5*::*SLC1-1 yjr116w∆* sphingolipid profiles very similar to those of 4∆ and 6∆ cells (not shown). The same was also found for such 5∆ strains having the non-deleted 6^th^ gene (YJR116w or YPR114w) behind a tet_off_ promoter and downregulated under doxycycline (not shown). On the side, the lipid analysis showed that in 4∆ and 6∆ cells the fatty acids of most glycerophospholipids (GPLs) including the mitochondrial cardiolipins have significantly more C atoms than in WT (Fig B, panel A in S1 File for phosphatidylinositol, and Fig C in S1 File). The inability of 4∆ cells to use C26-CoA for ceramide synthesis may lead to a stagnation and build up of intermediates in the fatty acid elongation pathway that conduces to the increased utilization of [C18-26]-CoAs for the biosynthesis of GPLs. Overall, our data confirm that 6∆ cells still make similar amounts of IPCs and MIPCs containing C26 fatty acids as 4∆ cells [14] and that they also contain similarly elevated amounts of sphingolipids with shorter fatty acids (32–38 species). Hence, these data argue that Ypr114w and Yjr116w are not involved in the alternative ceramide synthesis pathway.

### Cation hypersensitivity of *ypr114w∆* and *yjr116w∆* deletion strains

**Fig 3:**
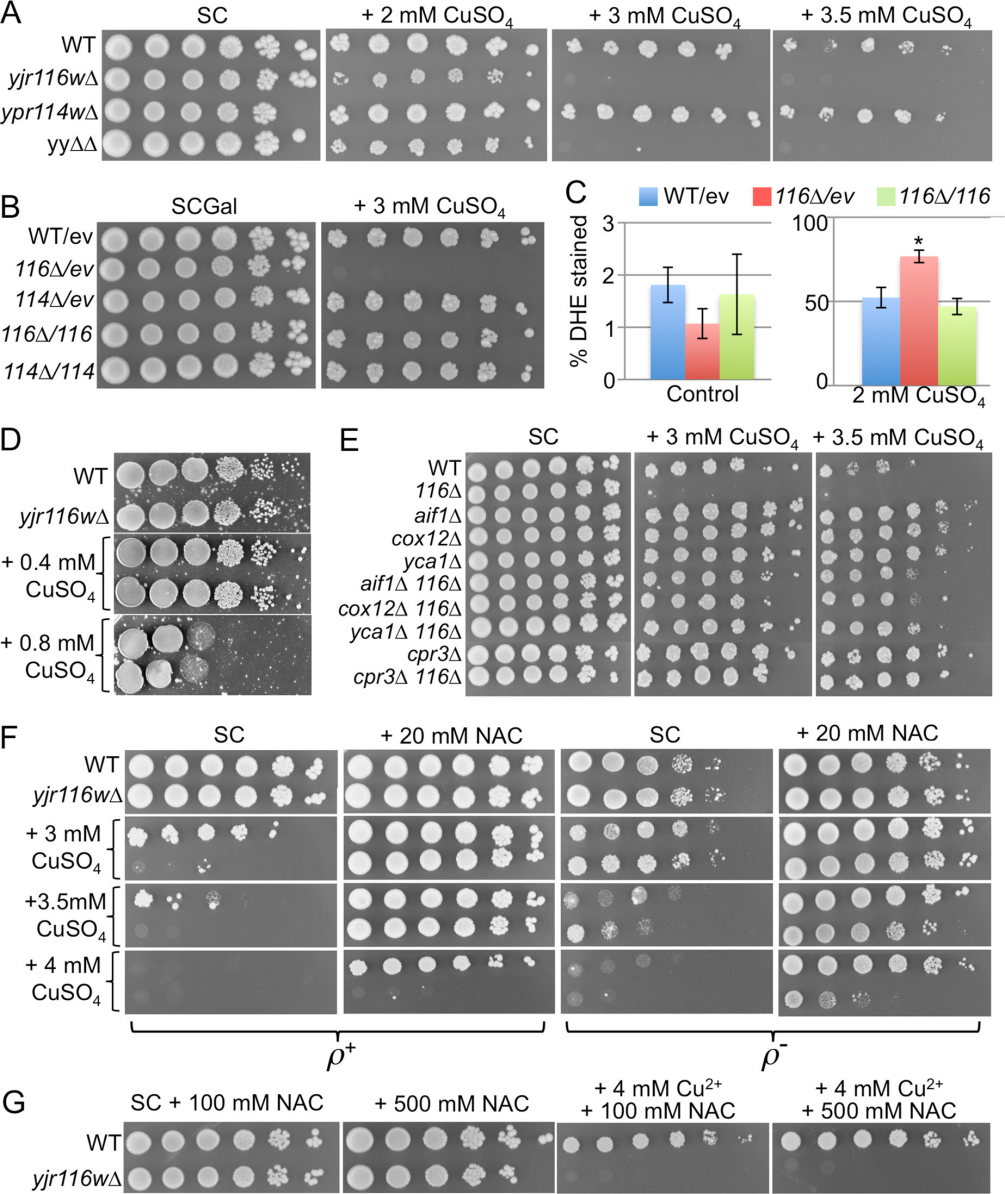
Disruption of YJR116w makes cells hypersensitive to copper. A. 10-fold serial dilutions of *yjr116w∆*, *ypr114w∆* and yy∆∆ mutants were plated on SC containing 2% glucose and indicated concentrations of CuSO_4_. B. WT, *yjr116w∆* (116∆), or *ypr114w∆* (114∆) cells harboring either empty pYES2NT vector (ev) or the same expressing YJR116w or YPR114w (114 or 116, respectively), were plated on SC with 2% galactose (Gal). C. Superoxide anion (O_2_^-^) levels of three independent clones of the same strains grown for 15 h in presence or absence of 2 mM CuSO_4_ were assessed by staining cells with DHE. * *yjr116w∆* cells showed a significantly higher percentage of DHE positive cells than WT (P = 0.0039). D. As A, but plates were incubated anaerobically and contained 5 mg ml^-1^ Tween 80 and 20 μg ml^-1^ ergosterol. The freckles are precipitates caused by these additions to the medium. In each plate section, in the upper and lower rows are WT and *yjr116w∆* cells, respectively. E. Copper sensitivity of indicated mutants was tested as in A. F. First and 3^rd^ columns as in A, the others supplemented additionally with 20 mM NAC. The 3^rd^ and 4^th^ columns show the growth behavior of ρ^*-*^ strains lacking the respiratory chain. G, growth on copper and higher concentrations of NAC.

Cocultivation of a homozygous diploid yeast deletion strain collection in presence of 1144 different chemical stresses in a high throughput effort showed that *ypr114w∆/ypr114w∆* are hypersensitive to 2 mM MnCl_2_, to 600 mM NaCl and to wiskostatin (29 μM), whereas they are hyperresistant to CoCl_2_ (1.25 mM), miconazole (100 μM), myriocin (0.2 μg ml^-1^), and H_2_O_2_ (5 mM)[23]. Similarly, in these comparative fitness assays *yjr116w∆/yjr116w∆* were found to be hypersensitive to Cu^2+^ (10 mM, P-value < 10^−12^), Zn^2+^ (1.9 mM), 1,7-octadiene (0.25%), diallyl disulfide (0.01%; affecting the cellular redox state [24]), norcantharidin (2.5 mM) and 1,4-dimethylendothall (100 μM), but hyperresistant to inositol- or paraaminobenzoic acid-free medium. In search of possible functions we tested whether we could reproduce some of these phenotypes under non-competitive growth conditions, i.e. in simple serial dilution plating assays. With regard to ions, *ypr114w∆*, *yjr116w∆* or yy∆∆ mutants showed no altered sensitivity to Ca^2+^, Mn^2+^, NaCl, Co^2+^ or Zn^2+^, Fe^2+^, in such assays, nor could we detect any hypersensitivity to low or high pH and low or high temperature (Fig D, panels A-H in S1 File). However, all mutant strains were slightly resistant to Cd^2+^ (Fig D, panel I in S1 File) and strains harboring *yjr116w∆* were found to be sensitive to Cu^2+^, whereby this hypersensitivity could be suppressed by reintroduction of *YJR116w* into the deletion strain (Fig 3A and 3B).

(As seen in Fig 3, WT as well as mutant cells often grew on copper as distinct heaps of cells, which did not seem to represent a preexisting minority of copper resistant cells as their frequency was the same over a wide range of dilutions). When *yjr116w∆* were crossed with the corresponding WT strain, Cu^2+^ hypersensitivity cosegregated in 12 out of 12 tetrads with the *yjr116w*::*KanMX* allele (Fig E, panel A in S1 File). Copper hypersensitivity of *yjr116w∆* was also seen in the W303 background and to a slightly lesser degree in the YPK9 background (Fig E, panel B in S1 File).

Copper can be in two oxidation states and causes the appearance of reactive oxygen species (ROS) which, when elevated above a critical level, can be detected by staining cells with dihydroethidium (DHE)[25]. In our hands DHE stainable cells usually appear only after 24 h when cells reach stationary phase, but copper apparently makes them appear earlier. Indeed, as measured by the frequency of DHE stained cells, CuSO_4_ treatment for 15 h caused higher superoxide accumulation in *yjr116w*∆ than WT (Fig 3C). In keeping with this, *yjr116w*∆ are no more sensitive to Cu^2+^ in anaerobic conditions (Fig 3D), although no cells grew anaerobically at higher than 0.8 mM Cu^2+^.

A seminal paper on copper mediated toxicity [25] reports that the deletions in certain, albeit not all genes required for mitochondrial respiration (*NDI1*, *QCR7*, *COX12*) and deletion of the mitochondrial peptidyl-prolyl cis-trans isomerase (*CPR3*) rescue cells from copper toxicity, whereas deletions of cytosolic apoptosis mediating factors such as *YCA1* or *AIF1* did not. To see if these genes also play a role in the copper hypersensitivity of *yjr116w∆* cells, we created the corresponding double mutants and tested their copper sensitivity as shown in Fig 3E. The data indicate that not only ablation of mitochondrial respiration, but also of mediators of apoptosis increase the resistance of cells towards copper both in WT as well as *yjr116w∆*. Experimental differences may explain the discrepancy with regard to *yca1∆* and *aif1∆* cells: Liang and Zhou measured cell survival, not growth, by exposing cells for 12 h to 6 mM Cu^2+^ in liquid culture and counting colony forming units thereafter. 8 mM Cu^2+^ were needed to kill all cells [25]. In our simple growth tests on plates, cells were continuously exposed to copper during 3 days and 4 mM Cu^2+^ was sufficient to abrogate cell growth in WT cells (Fig 3F, 1^st^ column). With regard to *yjr116w∆* cells, they were rescued to a significant extent by all suppressor deletions tested, but still grew less than WT cells carrying these suppressor deletions (Fig 3E). Thus, it appears that *yjr116w∆*, as WT cells, undergo a mitochondrially orchestrated type of apoptosis, but that *yjr116w∆* have an additional reason for being copper hypersensitive.

To test if ROS are involved in the oxygen-dependent copper hypersensitivity of *yjr116w∆* more directly we also plated the cells on N-acetyl-cysteine (NAC), a membrane permeating antioxidant elevating reduced glutathione levels. NAC significantly increased the copper resistance of WT and even more so of *yjr116w∆* cells, so that WT and *yjr116w∆* grew equally well in 3.5 mM Cu^2+^; yet, a difference remained in that WT grew in 4 mM Cu^2+^ when NAC was present, whereas *yjr116w∆* didn’t (Fig 3F, 2^nd^ column). The effect of NAC was due to its thiol group, as N-acetyl-serine had no effect (Fig E, panel C in S1 File). The copper hypersensitivity of *yjr116w∆* persisted even at very high concentrations of NAC (Fig 3G in S1 File). Thus, the copper hypersensitivity of *yjr116w∆* on NAC maybe due to a type of ROS that is not counteracted by NAC or by an abnormal copper hypersensitivity not related to ROS.

So-called petite or ρ^-^ cells lack mitochondrial respiration and, as *cox12∆*, showed a decreased apoptosis rate on copper in comparison to ρ^+^ cells [25]. In our growth assays ρ^-^ cells formed smaller colonies than ρ^+^ cells as expected, but WT ρ^-^ did not grow better on copper than the corresponding ρ^+^ strains (Fig 3F, 1^st^ vs. 3^rd^ column). In contrast, *yjr116w∆* ρ^-^ grew much better than *yjr116w∆* ρ^+^ and in fact grew as well as WT ρ^-^ up to 3.5 mM Cu^2+^ (Fig 3F, 3^rd^ column). Yet again, at 4 mM Cu^2+^, 20 mM NAC restored normal growth to WT ρ^-^, but rescued growth of *yjr116w∆* ρ^-^ cells only partially (Fig 3F, 3^rd^ and 4^th^ column). Thus, the copper hypersensitivity of *yjr116w∆* is complex and mainly due to an elevated mitochondrial ROS production in presence of copper (Fig 3C), but also a minor component that is independent of mitochondrial respiration, possibly due to extra-mitochondrially generated ROS that cannot be counteracted by NAC or due to some susceptibility unrelated to ROS. Data also clearly indicate that the toxicity of copper in WT cells may be mediated in part by ROS generated outside the mitochondria (Fig 3F, 3rd vs. 4th column). Nevertheless, at higher concentrations of Cu^2+^ (6 mM), WT ρ^-^ could not grow even with the help of very high concentrations of NAC (Fig E, panel D in S1 File).

In the cocultivation assays of [23], amongst about 5000 diploid deletion strains, 28 were even more Cu^2+^ hypersensitive than *yjr116w∆*. Amongst those 28, as expected, *cup2∆/cup2∆*, lacking the obligatory transcription factor inducing the metallothioneins Cup1 and Crs5 [26] as well as *csg1∆/csg1∆* and *csg2∆/csg2∆* strains, which lack the Golgi mannosyltransferases making MIPC. The latter were isolated as **c**alcium **s**ensitive **g**rowth (*csg*) mutants being unable to grow in 50 mM Ca^2+^ [27]. We tested *csg1∆*, *csg2∆* and some others amongst these 28 strains to see whether their hypersensitivity can be seen in growth assays and can be cured by NAC (Fig E, panel E in S1 File and Table D in S1 File). Interestingly, some of the tested mutants were similar to *yjr116w∆* in that they were only partially rescued by NAC (class C), but others were not rescued at all (class B)(Table D in S1 File). This illustrates that copper causes in many strains a ROS-independent kind of toxicity, which may account for part of the copper hypersensitivity of *yjr116w∆* and which may be related to the non-apoptotic death seen also in WT cells at very high copper concentrations [25]. Unexpectedly, mutations affecting the synthesis of mannosylated sphingolipids (*csg1∆*, *csg2∆*, *ipt1∆)* proved to be hyperresistant, even though they had been found to be hypersensitive in competitive growth assays [23]. The mechanisms behind the hyperresistance to Cu^2+^ observed in some, and the ROS-independent toxicity of Cu^2+^ in other strains (Fig E, panel E in S1 File) are presently unknown. It also is unclear why in the absence of oxygen WT cells cannot grow at higher than 0.8 mM Cu^2+^.

### *Ypr114w∆* are more resistant to serine palmitoyltransferase inhibitors than WT

**Fig 4:**
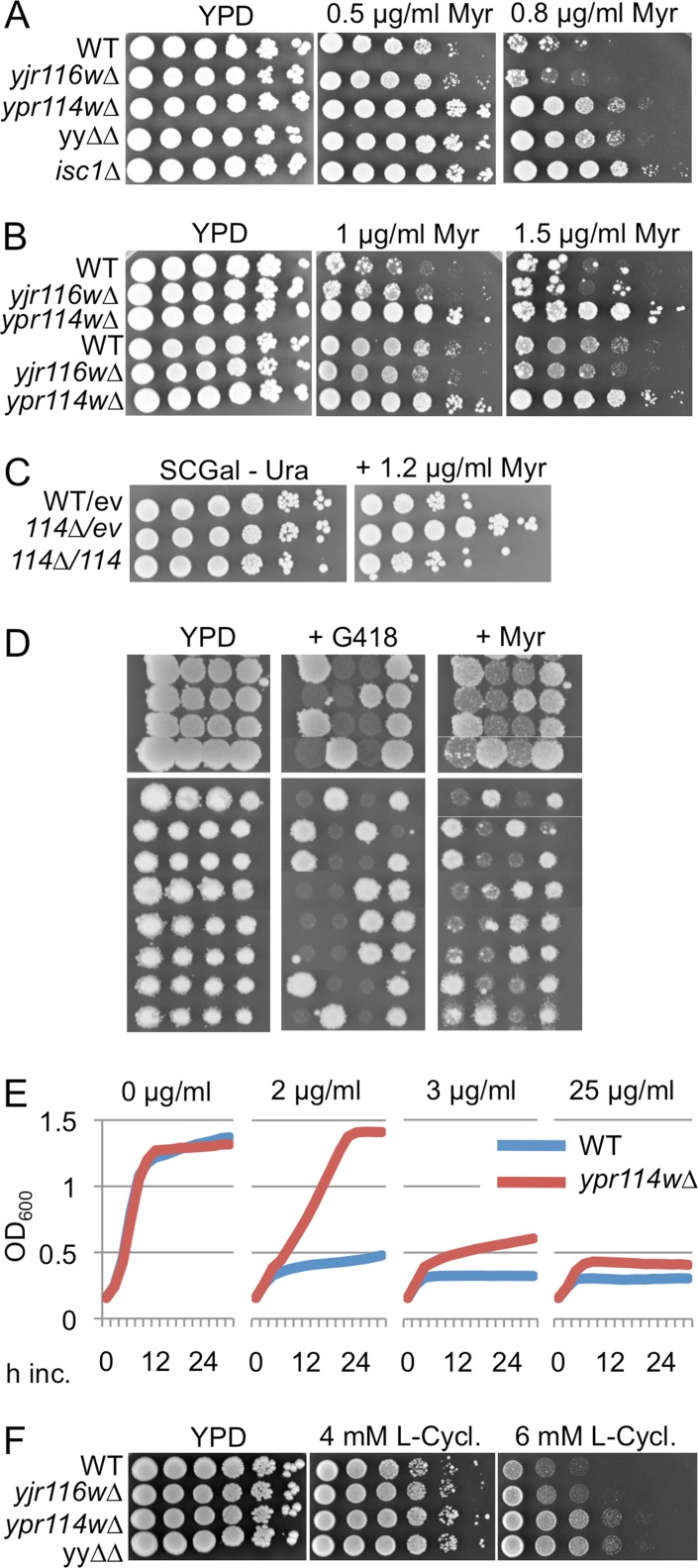
Disruption of YPR114w makes cells hyper-resistant to SPT inhibitors. A. WT and mutant strains were plated on YPD with or without myriocin (Myr) and incubated at 30°C for 3 days. B. As A, but the upper three rows show mutants in the W303 background, the lower three rows in the YPK9 background. C. As A, but *ypr114w∆* cells harboring empty vector pYES2NT (ev) or the same with YPR114w (114) were plated. D. WT B4742 was crossed with *ypr114w∆*, tetrads were dissected and its four spores aligned horizontally. After two days, the clones were replicated onto YPD media supplemented with nothing, G418 or myriocin (1.2 μg ml^-1^). E. Growth of WT = BY4742 and *ypr114w∆* in liquid YPD with concentrations of myriocin indicated at the top. F. As A, but with SPT inhibitor L-cycloserine.

Not being able to see in plain growth assays the reported NaCl and Mn^2+^ hypersensitivity of *ypr114w∆*, we tested other described hypersensitivities [23]. The very significant hypersensitivity of *ypr114w∆/ypr114w∆* to wiskostatin (P-value < 10^−12^), a drug causing a rapid, profound and irreversible decrease in cellular ATP in mammalian cells [28], was also not seen in growth assays (Fig F, panel A in S1 File), but we confirmed a distinct resistance of *ypr114w∆* and yy∆∆ to the serine palmitoyltransferase (SPT) inhibitor myriocin which is partially obfuscated by the frequent appearance of suppressors (Fig 1A; Fig 4A).

**Fig 5:**
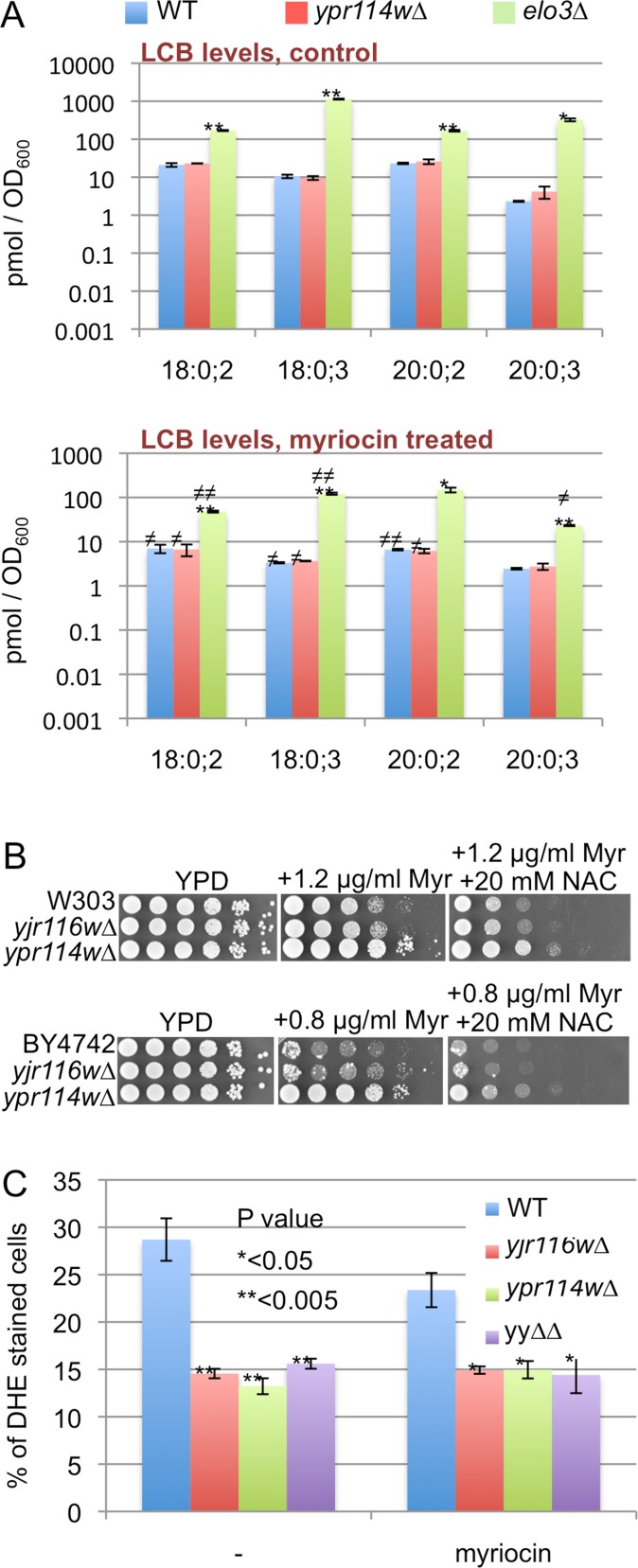
Effect of myriocin treatment on LCB levels. A. Cells growing exponentially in YPD were further incubated without (upper plot) or with 20 μg ml^-1^ myriocin (lower plot) for 4 hrs and LCB levels in the lipid extracts were determined by ESI-MS. A similar pretreatment with 2 μg ml^-1^ of myriocin repressed LCB levels of *ypr114w∆* and WT to the same degree, albeit less strongly (not shown). B. Plates containing myriocin with or without 20 mM NAC were incubated for 2 days (top sections W303 background, bottom sections BY4742 background). C. Strains in the W303 background were grown in YPD with or without myriocin (1.2 μg ml^-1^) for 24 h and superoxide anions were detected by staining with DHE. After 24 h all strains had grown to the same density.

As a control we used *isc1∆* cells, which lack the phospholipase C-like phosphodiesterase for complex sphingolipids and exhibit some of the phenotypes of *yjr116w∆* and *ypr114w∆* cells. Their predominant ceramide species in mitochondria (Cer44:0;4) after diauxic shift are drastically reduced, whereas Cer44:0;3 is correspondingly increased [29]. The myriocin resistance of *ypr114w∆* was also seen in two other genetic backgrounds and was abolished by the transfection of WT *YPR114w* into *ypr114w∆*, either using a multicopy vector on galactose (Fig 4B and 4C) or a centromeric (pBF775) vector on glucose (not shown). Fig 4C also demnostrates that overexpression of YPR114w does not induce myriocin hypersensitivity. Moreover, when *ypr114w∆* were crossed with the corresponding WT strain, myriocin resistance cosegregated in 12 out of 12 tetrads with the *ypr114w*::*KanMX* allele (Fig 4D). Myriocin resistance could also be observed in liquid media (Fig 4E). *Ypr114w∆* were also somewhat resistant to the less specific SPT inhibitor L-cycloserine that forms an irreversible adduct with pyridoxal 5’-phosphate, the coenzyme of SPT and several other enzymes [30](Fig 4F). One possible reason for increased resistance to SPT inhibitors would be a constitutively elevated SPT activity, which can be reflected by an increased sensitivity to LCBs such as PHS [9]. PHS sensitivity of all our mutants however was normal (Fig F, panel B in S1 File), as was sensitivity to aureobasidin A, an inhibitor of IPC synthase (Fig F, panel C in S1 File). We considered the possibility that myriocin would be taken up less efficiently by *ypr114w∆* than WT cells, resulting in a less severe depression of SPT. Direct measurement of LCB levels after treatment with myriocin did however not support this hypothesis (Fig 5A).

Thus, the myriocin resistance of *ypr114w∆* appears to be due to the ability of this strain to grow better than WT even when LCB levels are depressed.

**Fig 6:**
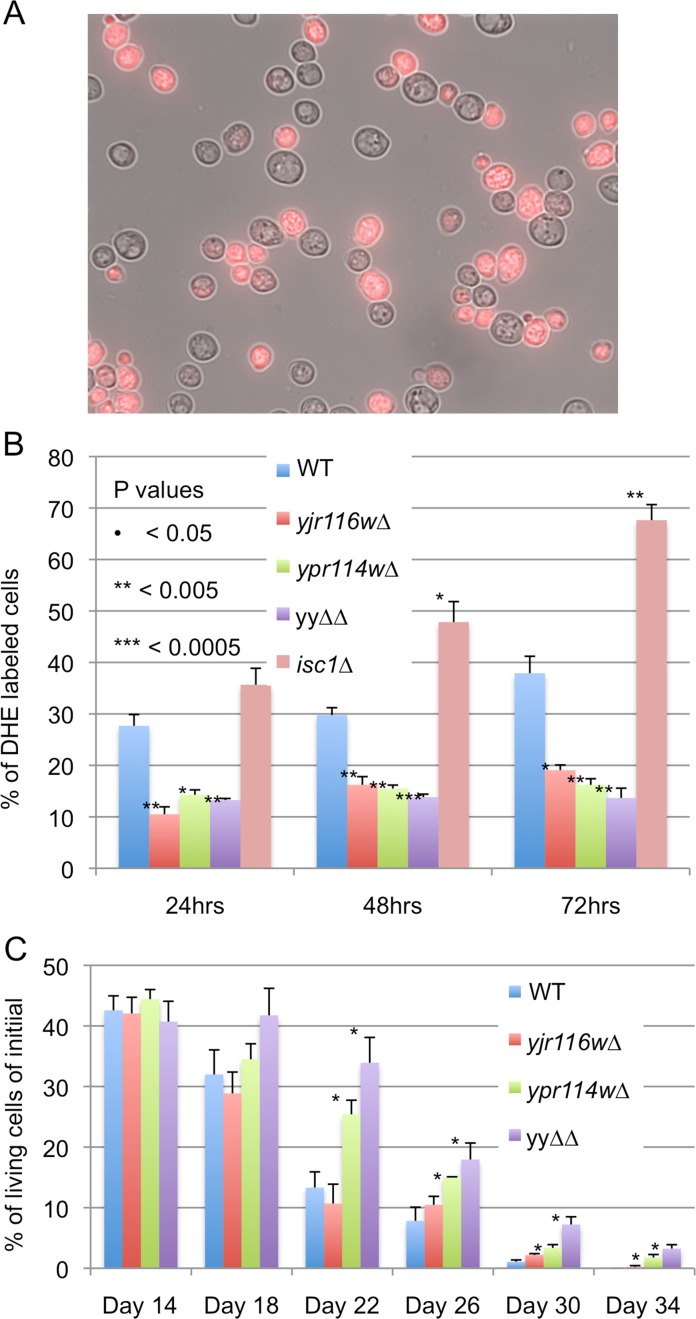
*Yjr116w∆* and *ypr114w∆* make lower amounts of superoxide anions in stationary phase than WT and have an increased CLS. A. Cells were stained with DHE for measurement of superoxide anion levels. Fluorescence and transillumination pictures were merged to illustrate the difference between positive and negative cells. B. Percentages of DHE positive cells amongst 200 cells were determined in three independent experiments and the difference between mutants and WT in the same condition was evaluated by student’s t-test with P< 0.05 = *, P< 0.005 = **. C. The chronological life spans were determined in diploid deletion strains by measuring the colony-forming units after different periods in water (see Materials and Methods). Data represent three biological replicates using different clones and the differences between mutants and WT were evaluated as above.

A recent study claimed that myriocin treatment was toxic to cells through the generation of ROS and that its toxicity could be overcome by adding 20 mM NAC to the nutrient agar [31]. We did not see any effect of NAC on the myriocin sensitivity of *ypr114w∆* or WT (Fig 5B). Also, we found that superoxide levels in stationary phase cells, as determined by the % of dihydroethidium (DHE) stained cells, were lower in *ypr114w∆*, *yjr116w∆* and yy∆∆ mutants than in WT cells and were not elevated by myriocin treatment in any cell type (Fig 5C). Others also had not observed an elevation of DHE staining in myriocin treated stationary phase cells [32]. Superoxide levels in our mutants were low also in the post-diauxic shift period, during which WT and *isc1∆* cells exhibited a steady increase (Fig 6A and 6B).

The increase in DHE staining cells was not seen because cells had died, since in several instances chronological aging in water produced more DHE stained cells than dead cells (Fig G in S1 File).

Further tests indicated that our mutants were not hypersensitive to rapamycin, calcofluor white (CFW) or tunicamycin and were able to induce normal amounts of the ER chaperon Kar2 in response to ER stress, but that they were slightly resistant to H_2_O_2_ (Fig H in S1 File).

ROS levels were found to increase during chronological aging and are considered to be a major factor limiting chronological life span (CLS)[33,34]. In keeping with their lower than normal superoxide levels, we observed a slight but distinct increase of CLS in *ypr114w∆* and yy∆∆ cells (Fig 6C).

### Import of proteins into the ER by *yjr116∆* and *ypr114∆* cells

**Fig 7:**
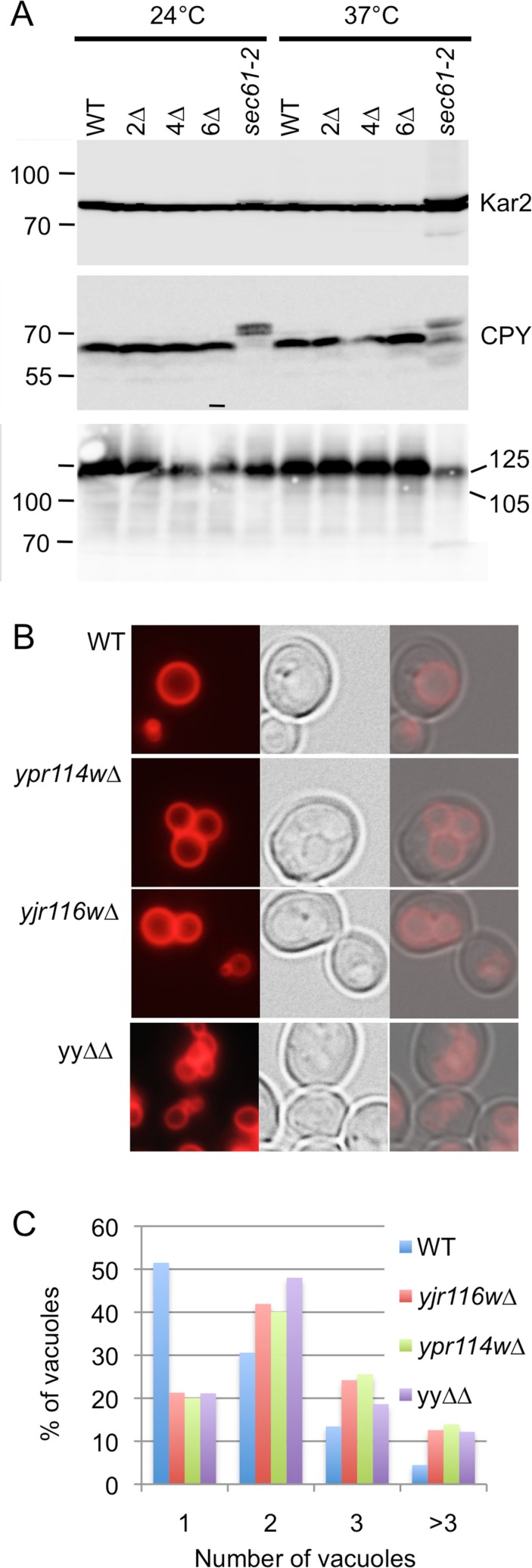
Protein targeting and organellar structures. A. Western blotting of cell extracts. Cells were grown to an OD_600_ of 0.8 either at 24°C or at 37°C. B and C. Vacuoles of cells growing in YPD were stained with FM4-64. Typical pictures are shown in B, the percentages of cells having 1 to >3 vacuoles are shown in C. >250 cells were inspected for each strain.

TRAM1, a mammalian paralog of Lag1 and Lac1, is required for the import of certain secretory proteins into the ER but deletion of Lag1 and Lac1 did not affect this process in vivo [7]. Given the homology of YPR114w and YJR116w with mammalian TRAM1 (Fig 1B), we decided to test whether these proteins play any role in ER import. As seen in Fig 7A, no cytosolic accumulation of any secretory protein could be observed in *yjr116∆*, *ypr114∆*, yy*∆∆* or 6∆ cells, which latter contains deletions in all known yeast TLC-domain proteins.

This suggests that the translocation process in yeast does not depend on any TRAM-like component.

Genetic evidence suggests that YJR116w helps the functionality of the proton pumping V-ATPase of the vacuole [35], the V1 complex of which requires C26:0-containing sphingolipids for proper function [36]. Yet, quinacrine, a fluorescent compound that accumulates in acidic compartments, was found to accumulate to normal levels in the vacuoles of our mutants and thus did not reveal any acidification defect (Fig I in S1 File). However, when staining cells with FM4-64, vacuoles of *yjr116*∆, *ypr114*∆ and yy∆∆ tended to be more fragmented than in WT (Fig 7B). The kinetics of endocytosis followed by FM4-64 internalization were however normal in all three backgrounds (not shown). We also surveyed the morphology of other intracellular organelles and found that ER, Golgi, plasma membrane, endosomes and mitochondria showed normal morphology (Fig J in S1 File, data not shown).

Unlike *isc1∆* cells [29], the mutants did not generate petite colonies at an elevated rate (Fig K in S1 File) and grew normally on nonfermentable carbon sources (not shown).

## Discussion

The main intent of our report was to put to the test our educated guess, namely that the *LAG1* homologs YJR116w and YPR114w would be responsible for residual ceramide and IPC synthesis in 4∆ cells [14]. Experiments say that the 4∆ and 6∆ cells contain the same amounts of ceramides, IPCs and MIPCs including low amounts of species with 44 C atoms. This leads to the conclusion that these residual sphingolipids are made neither by YJR116w nor by YPR114w.

Not finding any further Lag1 and Ypc1 homologs in yeast, it has to be envisaged that these very low amounts of sphingolipids may arise through a non-catalyzed attachment of fatty acids to LCBs. Acyl migration from GPLs to LCBs has been observed during lipid extraction with chloroform or during mild base treatment with NaOH [37] raising the possibility that the analogous reaction may generate IPCs from lyso-IPC (inositolphosphoryl-PHS), which indeed can be found in into 4∆ cells [14]. However, 4∆ cells don’t contain “lyso-MIPCs”, and we therefore argue that at least the MIPCs found in 4∆ and 6∆ (Fig B, panel D in S1 File) cannot arise during lipid extraction by an analogous acyl migration originating from GPLs. Thus, if non-enzymatic catalysis is at work, it has to be working *in vivo*. It is possible that present day ceramide synthases came into existence because they were able to accelarate a slow spontaneous condensation reaction. What is clear however is that the *in vivo* generation of small amounts of ceramides and their transformation into IPCs by Aur1 is necessary for the growth/survival of 4∆ cells, since *AUR1* remains essential in these cells and AbA blocks the growth of 4∆ cells [12,14].

A significant part of the copper hypersensitivity of *yjr116w∆* can be abolished by NAC but the NAC-resistant toxicity sets in at a lower [Cu^2+^] in these mutants than in WT. It was shown that free [Cu^2+^] in the cytosol, the species that may be responsible for ROS generation is zero as long as [Cu^2+^] in the growth medium does not reach toxic levels [38]. Thus, one possible explanation of the copper hypersensitivity of *yjr116w∆* would be that the levels of one of the copper-chelating proteins such as Cup1, Crs5, Lys8 or Sod1 is reduced in *yjr116w*∆ and ROS generation starts at a lower [Cu^2+^] in the medium than in WT. This would explain why ROS reach higher levels in presence of copper in *yjr116w∆* than in WT, although in exponentially growing cells, *yjr116w∆* has lower ROS levels than WT (Fig 3C vs. 5D). Copper hypersensitive mutants according to the data of [23] are significantly enriched in GO terms such as protein localization to Golgi apparatus [GO:0034067], endosomal transport [GO:0016197], vacuolar transport [GO:0007034] (not shown). Moreover, deletions of genes operating in many other processes also are resulting in copper hypersensitivity [23,39–41], so that there seem to be many processes influencing copper sensitivity and the precise origins of the multifaceted, largely oxygen-, respiration- and ROS-mediated copper sensitivity of *yjr116w*∆ for the moment remains unknown.

Sphingolipids have been proposed to regulate mitochondrial function. Thus, overexpression of *YDC1*, supposed to reduce ceramide levels, causes mitochondrial and vacuolar fragmentation and dysfunction [42]. Moreover, the classical mutant linking sphingolipids to altered mitochondrial ROS production is *isc1∆*, lacking the phospholipase C type hydrolase of IPCs, MIPCs and M(IP)_2_Cs. However, *isc1∆*, differently from *ypr114w∆*, shows abnormal mitochondrial morphology, generates petites at high frequency, does not grow well on nonfermentable carbon sources, has elevated ROS levels in the absence of stresses, is H_2_O_2_ hypersensitive and has a reduced CLS [29,43,44].

While our studies shed some light on abnormal stress responses or abnormalities of *ypr114w∆* and *yjr116w∆* cells, the exact function of these *LAG1* homologs remains to be elucidated. BIOGRID (http://thebiogrid.org) indicates only very few, namely 4 and 19 genetic interactions for *yjr116w∆* and 19 *ypr114w∆*, respectively. The strongest ones are negative interactions of *ypr114w∆* with *mmm1∆* and *mdm32∆*, Mmm1 being a subunit of the ERMES complex linking the ER to the mitochondrial outer membrane, Mdm32 being a mitochondrial inner membrane protein required for normal mitochondrial morphology. We generated *ypr114w∆ mmm1∆* and *ypr114w∆ mdm32∆* strains, but could not observe any synthetic growth defect in these double mutants on plates (data not shown).

Our study so far suggests that the scarcity of genetic interactions of *ypr114w*∆ and *yjr116w∆* reported in BIOGRID may not be the reflection of any functional redundancy. Indeed, in spite of their homology (Fig 1B), our data did not allow to identify any common function of YJR116w and YPR114w in that most phenotypes present in single mutants were not enhanced in yy∆∆, except CLS (Fig 6C).

Studies on CLN8, a human TLC domain protein lacking ceramide synthase activity which, when mutated, causes neurodenerative lipofuscinosis led to the proposal that it acts as sensor or regulator of lipid homeostasis, although through unknown molecular mechanisms [45]. Similarly, YJR116w and YPR114w may encode for sensors that play some regulatory roles in certain stress situations, which still must be frequent and strong enough to force the conservation of this type of proteins in the fungal taxa (Fig 1D).

## Supporting Information

S1 File (PDF)

## References

[1] Dickson RC, Sumanasekera C, Lester RL (2006) Functions and metabolism of sphingolipids in Saccharomyces cerevisiae. Prog Lipid Res 45: 447–465. 16730802 10.1016/j.plipres.2006.03.004

[2] Dickson RC (2010) Roles for sphingolipids in Saccharomyces cerevisiae. Adv Exp Med Biol 688: 217–231. 20919657 10.1007/978-1-4419-6741-1_15 PMC5612324

[3] Guillas I, Kirchman PA, Chuard R, Pfefferli M, Jiang JC, Jazwinski SM et al (2001) C26-CoA-dependent ceramide synthesis of Saccharomyces cerevisiae is operated by Lag1p and Lac1p. EMBO J 20: 2655–2665. 11387200 10.1093/emboj/20.11.2655 PMC125493

[4] Winter E, Ponting CP (2002) TRAM, LAG1 and CLN8: members of a novel family of lipid-sensing domains? Trends Biochem Sci 27: 381–383. 12151215 10.1016/s0968-0004(02)02154-0

[5] Vallee B, Riezman H (2005) Lip1p: a novel subunit of acyl-CoA ceramide synthase. EMBO J 24: 730–741. 15692566 10.1038/sj.emboj.7600562 PMC549621

[6] Jiang JC, Kirchman PA, Zagulski M, Hunt J, Jazwinski SM (1998) Homologs of the yeast longevity gene LAG1 in Caenorhabditis elegans and human. Genome Res 8: 1259–1272. 9872981 10.1101/gr.8.12.1259

[7] Barz WP, Walter P (1999) Two endoplasmic reticulum (ER) membrane proteins that facilitate ER-to-Golgi transport of glycosylphosphatidylinositol-anchored proteins. Mol Biol Cell 10: 1043–1059. 10198056 10.1091/mbc.10.4.1043 PMC25232

[8] Mao C, Xu R, Bielawska A, Obeid LM (2000) Cloning of an alkaline ceramidase from Saccharomyces cerevisiae. An enzyme with reverse (CoA-independent) ceramide synthase activity. J Biol Chem 275: 6876–6884. 10702247 10.1074/jbc.275.10.6876

[9] Mao C, Xu R, Bielawska A, Szulc ZM, Obeid LM (2000) Cloning and characterization of a Saccharomyces cerevisiae alkaline ceramidase with specificity for dihydroceramide. J Biol Chem 275: 31369–31378. 10900202 10.1074/jbc.M003683200

[10] Schorling S, Vallee B, Barz WP, Riezman H, Oesterhelt D (2001) Lag1p and Lac1p are essential for the Acyl-CoA-dependent ceramide synthase reaction in Saccharomyces cerevisae. Mol Biol Cell 12: 3417–3427. 11694577 10.1091/mbc.12.11.3417 PMC60264

[11] Jiang JC, Kirchman PA, Allen M, Jazwinski SM (2004) Suppressor analysis points to the subtle role of the LAG1 ceramide synthase gene in determining yeast longevity. Exp Gerontol 39: 999–1009. 15236759 10.1016/j.exger.2004.03.026

[12] Cerantola V, Guillas I, Roubaty C, Vionnet C, Uldry D, Knudsen J et al (2009) Aureobasidin A arrests growth of yeast cells through both ceramide intoxication and deprivation of essential inositolphosphorylceramides. Mol Microbiol 71: 1523–1537. 10.1111/j.1365-2958.2009.06628.x 19210614

[13] Voynova NS, Mallela SK, Vazquez HM, Cerantola V, Sonderegger M, Knudsen J et al (2014) Characterization of yeast mutants lacking alkaline ceramidases YPC1 and YDC1. FEMS Yeast Res 14: 776–788. 10.1111/1567-1364.12169 24866405

[14] Vionnet C, Roubaty C, Ejsing CS, Knudsen J, Conzelmann A (2011) Yeast cells lacking all known ceramide synthases continue to make complex sphingolipids and to incorporate ceramides into glycosylphosphatidylinositol (GPI) anchors. J Biol Chem 286: 6769–6779. 10.1074/jbc.M110.176875 21173150 PMC3057787

[15] Kageyama-Yahara N, Riezman H (2006) Transmembrane topology of ceramide synthase in yeast. Biochem J 398: 585–593. 16756512 10.1042/BJ20060697 PMC1559446

[16] Spassieva S, Seo JG, Jiang JC, Bielawski J, Alvarez-Vasquez F, Jazwinski SM et al (2006) Necessary role for the Lag1p motif in (dihydro)ceramide synthase activity. J Biol Chem 281: 33931–33938. 16951403 10.1074/jbc.M608092200

[17] Ejsing CS, Moehring T, Bahr U, Duchoslav E, Karas M, Simons K et al (2006) Collision-induced dissociation pathways of yeast sphingolipids and their molecular profiling in total lipid extracts: a study by quadrupole TOF and linear ion trap-orbitrap mass spectrometry. J Mass Spectrom 41: 372–389. 16498600 10.1002/jms.997

[18] Ejsing CS, Sampaio JL, Surendranath V, Duchoslav E, Ekroos K, Klemm RW et al (2009) Global analysis of the yeast lipidome by quantitative shotgun mass spectrometry. Proc Natl Acad Sci U S A 106: 2136–2141. 10.1073/pnas.0811700106 19174513 PMC2650121

[19] Sokol E, Almeida R, Hannibal-Bach HK, Kotowska D, Vogt J, Baumgart J et al (2013) Profiling of lipid species by normal-phase liquid chromatography, nanoelectrospray ionization, and ion trap-orbitrap mass spectrometry. Anal Biochem 443: 88–96. 10.1016/j.ab.2013.08.020 23994565

[20] Ejsing CS, Duchoslav E, Sampaio J, Simons K, Bonner R, Thiele C et al (2006) Automated identification and quantification of glycerophospholipid molecular species by multiple precursor ion scanning. Anal Chem 78: 6202–6214. 16944903 10.1021/ac060545x

[21] Kushnirov VV (2000) Rapid and reliable protein extraction from yeast. Yeast 16: 857–860. 10861908 10.1002/1097-0061(20000630)16:9<857::AID-YEA561>3.0.CO;2-B

[22] Quaranta D, Krans T, Espirito Santo C, Elowsky CG, Domaille DW, Chang CJ et al (2011) Mechanisms of contact-mediated killing of yeast cells on dry metallic copper surfaces. Appl Environ Microbiol 77: 416–426. 10.1128/AEM.01704-10 21097600 PMC3020553

[23] Hillenmeyer ME, Fung E, Wildenhain J, Pierce SE, Hoon S, Lee W et al (2008) The chemical genomic portrait of yeast: uncovering a phenotype for all genes. Science 320: 362–365. 10.1126/science.1150021 18420932 PMC2794835

[24] Lemar KM, Aon MA, Cortassa S, O'Rourke B, Muller CT, Lloyd D (2007) Diallyl disulphide depletes glutathione in Candida albicans: oxidative stress-mediated cell death studied by two-photon microscopy. Yeast 24: 695–706. 17534841 10.1002/yea.1503 PMC2292485

[25] Liang Q, Zhou B (2007) Copper and manganese induce yeast apoptosis via different pathways. Mol Biol Cell 18: 4741–4749. 17881727 10.1091/mbc.E07-05-0431 PMC2096605

[26] Culotta VC, Howard WR, Liu XF (1994) CRS5 encodes a metallothionein-like protein in Saccharomyces cerevisiae. J Biol Chem 269: 25295–25302. 7929222

[27] Beeler TJ, Fu D, Rivera J, Monaghan E, Gable K, Dunn TM (1997) SUR1 (CSG1/BCL21), a gene necessary for growth of Saccharomyces cerevisiae in the presence of high Ca2+ concentrations at 37 degrees C, is required for mannosylation of inositolphosphorylceramide. Mol Gen Genet 255: 570–579. 9323360 10.1007/s004380050530

[28] Guerriero CJ, Weisz OA (2007) N-WASP inhibitor wiskostatin nonselectively perturbs membrane transport by decreasing cellular ATP levels. Am J Physiol Cell Physiol 292: C1562–6. 17092993 10.1152/ajpcell.00426.2006

[29] Kitagaki H, Cowart LA, Matmati N, Vaena de Avalos S, Novgorodov SA, Zeidan YH et al (2007) Isc1 regulates sphingolipid metabolism in yeast mitochondria. Biochim Biophys Acta 1768: 2849–2861. 17880915 10.1016/j.bbamem.2007.07.019 PMC2121593

[30] Ikushiro H, Hayashi H, Kagamiyama H (2004) Reactions of serine palmitoyltransferase with serine and molecular mechanisms of the actions of serine derivatives as inhibitors. Biochemistry 43: 1082–1092. 14744154 10.1021/bi035706v

[31] Niles BJ, Joslin AC, Fresques T, Powers T (2014) TOR complex 2-Ypk1 signaling maintains sphingolipid homeostasis by sensing and regulating ROS accumulation. Cell Rep 6: 541–552. 10.1016/j.celrep.2013.12.040 24462291 PMC3985744

[32] Huang X, Liu J, Withers BR, Samide AJ, Leggas M, Dickson RC (2013) Reducing signs of aging and increasing lifespan by drug synergy. Aging Cell 12: 652–660. 10.1111/acel.12090 23601176 PMC3714353

[33] Farrugia G, Balzan R (2012) Oxidative stress and programmed cell death in yeast. Front Oncol 2: 64 10.3389/fonc.2012.00064 22737670 PMC3380282

[34] Herker E, Jungwirth H, Lehmann KA, Maldener C, Frohlich KU, Wissing S et al (2004) Chronological aging leads to apoptosis in yeast. J Cell Biol 164: 501–507. 14970189 10.1083/jcb.200310014 PMC2171996

[35] Finnigan GC, Ryan M, Stevens TH (2011) A genome-wide enhancer screen implicates sphingolipid composition in vacuolar ATPase function in Saccharomyces cerevisiae. Genetics 187: 771–783. 10.1534/genetics.110.125567 21196517 PMC3063671

[36] Chung JH, Lester RL, Dickson RC (2003) Sphingolipid requirement for generation of a functional v1 component of the vacuolar ATPase. J Biol Chem 278: 28872–28881. 12746460 10.1074/jbc.M300943200

[37] Luttgeharm KD, Cahoon EB, Markham JE (2015) A mass spectrometry-based method for the assay of ceramide synthase substrate specificity. Anal Biochem 478: 96–101. 10.1016/j.ab.2015.02.016 25725359

[38] Rae TD, Schmidt PJ, Pufahl RA, Culotta VC, O'Halloran TV (1999) Undetectable intracellular free copper: the requirement of a copper chaperone for superoxide dismutase. Science 284: 805–808. 10221913 10.1126/science.284.5415.805

[39] Entian KD, Schuster T, Hegemann JH, Becher D, Feldmann H, Guldener U et al (1999) Functional analysis of 150 deletion mutants in Saccharomyces cerevisiae by a systematic approach. Mol Gen Genet 262: 683–702. 10628851 10.1007/pl00013817

[40] Szczypka MS, Zhu Z, Silar P, Thiele DJ (1997) Saccharomyces cerevisiae mutants altered in vacuole function are defective in copper detoxification and iron-responsive gene transcription. Yeast 13: 1423–1435. 9434348 10.1002/(SICI)1097-0061(199712)13:15<1423::AID-YEA190>3.0.CO;2-C

[41] Blaiseau PL, Lesuisse E, Camadro JM (2001) Aft2p, a novel iron-regulated transcription activator that modulates, with Aft1p, intracellular iron use and resistance to oxidative stress in yeast. J Biol Chem 276: 34221–34226. 11448968 10.1074/jbc.M104987200

[42] Aerts AM, Zabrocki P, Francois IE, Carmona-Gutierrez D, Govaert G, Mao C et al (2008) Ydc1p ceramidase triggers organelle fragmentation, apoptosis and accelerated ageing in yeast. Cell Mol Life Sci 65: 1933–1942. 10.1007/s00018-008-8129-8 18500447 PMC11131899

[43] Rego A, Costa M, Chaves SR, Matmati N, Pereira H, Sousa MJ et al (2012) Modulation of mitochondrial outer membrane permeabilization and apoptosis by ceramide metabolism. PLoS One 7: e48571 10.1371/journal.pone.0048571 23226203 PMC3511487

[44] Rego A, Trindade D, Chaves SR, Manon S, Costa V, Sousa MJ et al (2014) The yeast model system as a tool towards the understanding of apoptosis regulation by sphingolipids. FEMS Yeast Res 14: 160–178. 10.1111/1567-1364.12096 24103214

[45] Carcel-Trullols J, Kovacs AD, Pearce DA (2015) Cell biology of the NCL proteins: What they do and don't do. Biochim Biophys Acta10.1016/j.bbadis.2015.04.027 25962910

