# Supplementary material for: Functions of Ceramide Synthase Paralogs YPR114w and YJR116w of Saccharomyces cerevisiae
Source: PLoS One. 2016 Jan 11;11(1):e0145831. doi: 10.1371/journal.pone.0145831 (PMC4713442; doi:10.1371/journal.pone.0145831)
Supplement: S1 File — (PDF) [file pone.0145831.s001.pdf]

S1 File. Supporting information for

Functions of ceramide synthase paralogs YPR114w and YJR116w of *Saccharomyces cerevisiae*

Supplemental Materials and Methods

Construction of strains

Deletion strains were constructed by using PCR derived deletion cassettes. For FBY7440, *SLC1-1* behind the *MET17* promoter was amplified from plasmid pBF212 using primers 5'-ATCGATTGCTCCAAAAAGGGACATAGCACACCGACAGACCcggatgcaagggttcgaatc -3' (Forward) and 5'-AAAAAGCTAAATAAAAGCGTTCCTTATCAGATATTACTCAggagggcgtgaatgtaagcg -3' (Reverse) having homology to *TRP5* up- and downstream regions (in caps) and the amplified product was transformed into the 4Δ. pBM150:LAG1 (FBY1184) strain to get 4Δ.LAG1 *trp5*::*SLC1-1* (FBY7436). Then the strain was grown on medium with 5'-fluoroanthranilic acid (FAA)[1] except that the medium contained 4% glucose and 1% galactose instead of 5% glucose. The obtained colonies were grown in Lester medium (LM) supplemented with Aureobasidin A to get rid of pBM150:LAG1 and obtain FBY7440. (5'-fluoroorotic acid does not select against *URA3* plasmids on LM).

FBY7471 was constructed as follows: The spHIS5 cassette was amplified from plasmid pUG27 using primers 5'-AAAAAAAAACAATATTTTTAAGCTATCGCTCTTGCCGCAcagctgaagcttcgtacgc -3' (Forward) and 5'-GTAAGGCGTTTCGTTGTTGAAGATAGAAAAAAAGGTAGGTgcataggccactagtggatctg -3' (Reverse) having homology to *YJR116w* up- and down-stream regions (in caps) and the PCR product was transformed into FBY7436 to obtain strain FBY7437. FBY7437 was then transformed with a hygromycin cassette amplified from plasmid

pFA6a-hphNT1 using primers 5'-GGAATTCAAGCACGAAGGTGAACATTGCCTACAAGAAAAAcgtacgctgcaggtcga -3' (Forward) and 5'-TATACACATATATGCACATGTTAAAGTGTGCCTCCATGGAatcgatgaattcgagctcg -3' (Reverse) having homology to *YPR114w* up- and downstream regions (in caps) to obtain strain FBY7468. The pBM150:LAG1 plasmid was shuffled out of the strain FBY7468 to get FBY7471.To generate the homozygous diploid WT, *yjr116w*Δ, *ypr114w*Δ and yyΔΔ strains FBY7483, FBY7484, FBY7485 and FBY7486, haploid *MAT***a** and *MAT*α strains of the same genotype in BY4741 and BY4742 backgrounds, respectively, were crossed with each other and selected on medium without Met, Lys and Leu and supplemented with G418, except for diploid WT.

To make petite strains, the cells were grown to exponential phase (OD_{600} = 0.8) in YPD, 2 μg ml^{-1} ethidium bromide was added and cells were further incubated at 30 °C. After 6 h, cells were washed twice with sterile distilled water and plated on YPD and incubated at 30°C. After a few days, colonies were plated on YPD and YP glycerol plates to confirm them as ρ^- strains.

Inositol labeling

Metabolic labeling with [^{3}H]*myo*-inositol was carried out as described [2]. Briefly, WT, 4Δ and 6Δ strains were grown in inositol-free LM medium at 24°C. Exponentially growing cells were collected and labeled in inositol-free LM medium with [^{3}H]*myo*-inositol at 24 °C for 2 h. Lipids were extracted, deacylated, desalted by Folch partitioning and analyzed by TLC in solvent 1 (chloroform:methanol:0.25% KCl, 55:45:10). For deacylation, samples were resuspended in 200 μl of solvent 2 (chloroform:methanol:water, 10:10:3) and 40 μl of 0.6 M NaOH was added, nothing to controls. The control samples were incubated on ice and samples to be deacylated at 37°C for 1 h. Thereafter 40 μl of 0.8 N acetic acid was added to all tubes and 40 μl of 0.6 M NaOH was added only to the control tubes.

For GPI anchor remodeling cells were labeled with [^{3}H]*myo*-inositol as above, but for 4 h at 30 °C. GPI anchor lipids were isolated and analyzed as described [3] and separated on

TLC in solvent 1. TLC plates were then exposed and visualized using a phosphorimager (Bio-Rad Laboratories, Hercules, CA).

Vacuolar acidification

To asses vacuolar acidification, 3 OD_{600} units of exponentially growing cells were taken and resuspended in staining buffer (100 mM HEPES pH 7.6, and 0.2% glucose, 200 μM quinacrine) incubated at 30°C for 10 min, centrifuged, washed twice with wash buffer (0.2% glucose in 100 mM HEPES, pH 7.6) and resuspended in ice cold wash buffer. Cells were viewed under an Olympus BX54 microscope equipped with a piezo-positioner using the FITC filter within less than 60 min.

Supplemental Tables

Table A. Sequences used for the cladogram in Fig 1D

**Query (*S.cerevisiae*)**	**Sequence obtained (Accession number)**	**Organism (Fungi)**	**Score first query/ score second query**	**Identity (%)**
LAG1/LAC1	EDP49621.1	*A.fumigatus*	588/586	47/45
LAG1/ LAC1	XP_001394107.1	*A.niger*	571/563	49/46
LAG1/LAC1	XP_956995.1	*N.crassa*	604/597	43/44
LAG1/LAC1	NP_982955.1	*A.gossypii*	554/554	68/64
LAG1/LAC1	XP_452132.1	*K.lactis*	530/539	65/64
LAG1/LAC1	XP_505079.1	*Y.lipolytica*	538/537	46/47
LAG1/LAC1	XP_717940.1	*C.albicans*	544/539	55/54
LAG1/LAC1	NP_596102.1	*S.pombe*	444/445	41/40
LAG1/LAC1	KDQ12730.1	*B.botryosum*	381/377	32/34
LAG1/LAC1	XP_007337961.1	*A.delicata*	379/367	31/34
YPR114w/YJR116w	KDQ21753.1	*B.botryosum*	206/225	22/26
YPR114w/YJR116w	XP_007347861.1	*A.delicata*	192/187	28/28
YPR114w/YJR116w	XP_453728.1	*K.lactis*	304/197	47/23
YPR114w/YJR116w	NP_982626.1	*A.gossypii*	271/197	38/25
YPR114w/YJR116w	EHA19120.1	*A.niger*	296/298	25/26
YPR114w/YJR116w	KMK57131.1	*A.fumigatus*	295/303	21/28
YPR114w/YJR116w	XP_965364.2	*N.crassa*	289/315	20/25
YPR114w/YJR116w	NP_594236.1	*S.pombe*	233/245	25/27
YPR114w/YJR116w	AEY95224.1	*A.gossypii*	187/250	24/43
YPR114w/YJR116w	XP_453546.1	*K.lactis*	201/266	27/49
YPR114w/YJR116w	KGR03554.1	*C.albicans*	229/265	27/34
YPR114w/YJR116w	XP_500400.1	*Y.lipolytica*	216/232	22/34

Homologues of *LAG1, LAC1,* YJR116w and YPR114w in fungal model organisms were identified by PSI-Blast (http://blast.ncbi.nlm.nih.gov/) in fungal taxid (taxid:4751) with default parameters (PSI-BLAST threshold 0.005, and two reiterations) using the query genes indicated in the first column. It appears that when defining homologs as sequences identified with a significance threshold of $P < 0.005$, each of the hits found with *LAG1* was equally found with *LAC1*, but not by YPR114w or YJR116w, and *vice versa*, that each hit found with YPR114w was also found with YJR116w but not *LAG1* or *LAC1*. The homologs are identified by their accession numbers and have all been used to create the cladogram of Fig 1D.

Table B. Yeast *Saccharomyces cerevisiae* strains.

Strain name	**Genotype**	**Reference**
WT (BY4742)	*MATα his3Δ1 leu2Δ0 lys2Δ0 ura3Δ0*	EUROSCARF, (Frankfurt, GE)
yjr116wΔ (FBY7540)	BY4742, but *yjr116wΔ::kanMX4*	Roger Schneiter
ypr114wΔ (FBY7541)	BY4742, but *ypr114wΔ::kanMX4*	Roger Schneiter
yyΔΔ (FBY4352)	BY4742, but *yjr116wΔ::kanMX4 ypr114wΔ::LEU2*	Vikram Ghugtyal
isc1Δ (FBY3335)	BY4741, *MAT***a** *his3Δ1 leu2Δ0 met15Δ0 ura3Δ0 isc1Δ::natMX*	This study
WT (W303-1A)	*MAT***a** *ade2-1 can1-100 ura3-1 leu2-3,112 trp1-1 his3-11,15*	EUROSCARF
4Δ.LAG1 (FBY1184)	W303-1A, but *lac1Δ::ADE2 lag1Δ::TRP1 ydc1Δ::natMX ypc1Δ::kanMX4* containing pBM150:LAG1	Vanessa Cerantola
4Δ (FBY7440)	W303-1A, but *lac1Δ::ADE2 lag1Δ::TRP1 ydc1Δ::natMX ypc1Δ::kanMX4 trp5Δ::SLC1-1*	This study
6Δ (FBY7471)	FBY7440, but *yjr116wΔ::spHIS5 ypr114wΔ::HYG*	This study
4Δ.LAG1 (FBY7436)	W303-1A, but *lac1Δ::ADE2 lag1Δ::TRP1, ydc1Δ::natMX ypc1Δ::kanMX4 trp5Δ::SLC1-1* containing pBM150:LAG1	This study
5Δ.LAG1 (FBY7437)	FBY7436, but *yjr116wΔ::spHIS5*	This study
6Δ.LAG1 (FBY7468)	FBY7437, but *ypr114wΔ::HYG*	This study
WT (BY4741)	*MAT***a** *his3Δ1, leu2Δ0, met15Δ0, ura3Δ0*	EUROSCARF
yjr116wΔ (FBY7538)	BY4741, but *yjr116wΔ::kanMX4*	EUROSCARF
ypr114wΔ (FBY7539)	BY4741, but *ypr114w::kanMX4*	EUROSCARF
yyΔΔ (FBY7482)	BY4741, but *yjr116wΔ::kanMX4 ypr114wΔ::LEU2*	This study
WT (FBY7443)	BY4741 *MAT***a** *his3Δ1 leu2Δ0 met15Δ0 ura3Δ0*, but containing pYES2/NT B	This study
yjr116wΔ (FBY7446)	FBY7443, but *yjr116wΔ::kanMX4*	This study
ypr114wΔ (FBY7447)	FBY7443, but *ypr114wΔ::kanMX4*	This study
116Δ.116 (FBY7444)	As FBY7446, but containing pBF771	This study
114Δ.114	As FBY7447, but containing pBF772	This study

(FBY7445)		
hac1Δ (YJU 82)	*MAT***α** *trp1-1 his3-Δ200 ura3-52 lys2-801 leu2-3,112 hac1Δ::TRP1*	[4]
WT (W303-1B)	*MATα ade2-1 can1-100 ura3-1 leu2-3,112 trp1-1 his3-11,15*	EUROSCARF
yjr116wΔ (FBY7497)	W303-1B, but *yjr116wΔ::spHIS5*	This study
ypr114wΔ (FBY7498)	W303-1B, but *ypr114wΔ::spHIS5*	This study
WT.mtGFP (FBY7499)	As BY4742, but containing pYES-mtGFP	This study
yjr116wΔ.mtGFP (FBY7500)	As yjr116wΔa (FBY7540), but containing pYES-mtGFP	This study
ypr114wΔ.mtGFP (FBY7501)	As ypr114wΔa (FBY7541), but containing pYES-mtGFP	This study
yyΔΔ.mtGFP (FBY7502)	As FBY4352, but containing pYES-mtGFP	This study
isc1Δ.mtGFP (FBY7503)	As FBY3335, but containing pYES-mtGFP	This study
WT (FBY7527)	As BY4742, but containing pBF776	This study
yjr116wΔ (FBY7528)	As yjr116wΔ (FBY7540), but containing pBF776	This study
ypr114wΔ (FBY7529)	As YPR114w (FBY7541), but containing pBF776	This study
116Δ.116 (FBY7530)	As yjr116wΔ (FBY7540), but containing pBF774	This study
114Δ.114 (FBY7531)	As ypr114wΔ (FBY7541), but containing pBF775	This study
mmm1Δ ypr114wΔ (FBY7496)	BY4741, but *mmm1Δ::kanMX ypr114wΔ::spHIS5*	This study
mdm32Δ ypr114wΔ (FBY7495)	BY4741, but *mdm32Δ::kanMX ypr114wΔ::spHIS5*	This study
WT (YPK9)	*MAT***a** *ade2-101^{ochre} his3-Δ200 leu2-Δ1 lys2-801^{amber} trp1-Δ63 ura3-52*	Michal Jazwinski
yjr116wΔ (FBY7491)	YPK9, but *yjr116wΔ::spHIS5*	This study
ypr114wΔ (FBY7492)	YPK9, but *ypr114wΔ::spHIS5*	This study
WT (FBY7483)	*MAT***a/α** *his3Δ1 / his3Δ1, leu2Δ0 / leu2Δ0, lys2Δ0 /LYS2, MET15 / met15Δ0, ura3Δ0 / ura3Δ0*	This study
yjr116wΔ (FBY7484)	As FBY7483, but *yjr116wΔ::kanMX / yjr116wΔ::kanMX*	This study
ypr114wΔ (FBY7485)	As FBY7483, but *ypr114wΔ::kanMX / ypr114wΔ::kanMX*	This study

yyΔΔ (FBY7486)	As FBY7483, but *yjr116wΔ::kanMX / yjr116wΔ::kanMX, ypr114wΔ::LEU2 / ypr114wΔ::LEU2*	This study
WT.SEC7-GFP (FBY7506)	As BY4742, but containing pSEC7-GFP	This study
yyΔΔ.SEC7-GFP (FBY7513)	As FBY4352, but containing pSEC7-GFP	This study
WT.SED5-GFP (FBY7505)	As BY4742, but containing pGFP-SED5	This study
yyΔΔ.SED5-GFP (FBY7512)	As FBY4352, but containing pGFP-SED5	This study
WT.SNC1-GFP (FBY7507)	As BY4742, but containing pGS416-SNC1	This study
yyΔΔ.SNC1-GFP (FBY7514)	As FBY4352, but containing pGS416-SNC1	This study
WT.SSO1-GFP (FBY7508)	As BY4742, but containing pSSO416	This study
yyΔΔ.SSO1-GFP (FBY7515)	As FBY4352, but containing pSSO416	This study
cpr3Δ yjr116wΔ (FBY7532)	*cpr3::kanMX yjr116wΔ::LEU2*	This study
aif1Δ yjr116wΔ (FBY7533)	*aif1Δ::kanMX yjr116wΔ::spHIS5*	This study
yca1Δ yjr116wΔ (FBY7532)	*yca1Δ::kanMX yjr116wΔ::spHIS5*	This study
cox12Δ yjr116wΔ (FBY7532)	*cox12Δ::kanMX yjr116wΔ::spHIS5*	This study
WT (FBY7536)	BY4742, but petite	This study
yjr116wΔ (FBY7537)	YJR116w (FBY7540), but petite	This study
WT (RH3435)	S288c background, *MAT***a** *his4 leu2 ura3 lys2 bar1*	Howard Riezman
yjr116wΔ (FBY7487)	RH3435, but *yjr116wΔ::URA3*	This study
ypr114wΔ (FBY7488)	RH3435, but *ypr114wΔ::URA3*	This study

Several strains are named *yjr116w*Δ or *ypr114w*Δ. In the manuscript, the corresponding control strain usually tells, which strain was used except for deletions with different mating types in the same genetic background, e.g. FBY7540 and FBY7538.
Single deletion strains not listed in this table were from the EUROSCARF collection.

Table C. Plasmids

pBM150:LAG1	*LAG1* in CEN ARS *URA3*, *GAL* promoter	[5]
pBF212	*SLC1-1* in p413 CEN ARS *HIS3, MET25* promoter	[6]
pYES2/NT B	2μ *URA3*, *GAL* promoter	Invitrogen
pBF771	*YJR116w* in pYES2/NT B	This study
pBF772	*YPR114w* in pYES2/NT B 2μ	This study
pYES-mtGFP	mtGFP in pYES2/NT B 2μ	[7]
pBF776	pCM172 (CEN *TRP1*, tet_{off} promoter) from EUROSCARF, but having also *LEU2*	This study
pBF775	*YPR114w* in pBF776	This study
pSEC7-GFP	*SEC7-GFP* in CEN *URA3*, *ADH1* promoter http://www2.brc.riken.jp/lab/dna/detail.cgi?rdbno=08662	Akihiko Nakano
pGFP-SED5	GFP-*SED5* in CEN *URA3, ADH1* promoter http://www2.brc.riken.jp/cache/dna/8658	Akihiko Nakano
pGS416-SNC1	GFP-*SNC1* in CEN *URA3*, *TPI1* promoter	[8] H. Pelham
pSSO416	GFP-*SSO1* in CEN *URA3, TPI1* promoter	F. Reggiori
pPHM5416	GFP-*PHM5* in CEN *URA3, TPI1* promoter	[9]
pUG27	loxP-P_{AgTEF1}-**Sphis5-**T_{AgTEF1}-loxP, http://web.uni-frankfurt.de/fb15/mikro/euroscarf/data/pUG27.html	EUROSCARF
pFA6a-hphNT1	hph gene from Klebsiella pneumonia (for hygromycin B resistance) http://web.uni-frankfurt.de/fb15/mikro/euroscarf/data/pFA6hphNT1.html	EUROSCARF

Table D. Modulation of copper sensitivity by N-acetyl-L-cysteine (NAC)

**Strain**	**Resistance to copper**	**Improved by NAC**	**Category**
BY4741	As WT	Yes, as WT	A
*yps6*Δ	↓↓	No	B
*hac1*Δ	↓↓	Yes, as WT	A
*vps8*Δ	↓↓	Yes, but less than WT	C
*csg2*Δ	↑↑	NA[2)]	D
*ipt1*Δ	↑↑	NA	D
*vps60*Δ	as WT	Yes, as WT	A
*erg4*Δ	as WT	Yes, but less than WT	E
*cup2*Δ	↓↓	No	B
*psd2*Δ	as WT	Yes, as WT	A
*vps35*Δ	as WT	Yes, but less than WT	E
*cnb1*Δ	↓↓	No	B (C)
*mid1*Δ	↓↓	No	B
*csg1*Δ	↑↑	Yes, as WT	D
taz1	↓↓	No	B (C)
*aqy1*Δ	↓↓	No	B

The growth behavior of each strain in Fig E, panel E was compared to the one of WT and this allowed classifying strains into phenotypes A - E defined as follows:

A: Sensitivity in presence and absence of NAC as in WT

B: Hypersensitive, NAC cannot rescue hypersensitivity

C: Hypersensitive, NAC rescues hypersensitivity only partially

D: Hyperresistant

E: Sensitivity in absence of NAC as in WT, but more sensitive than WT in presence of NAC

[1)] ↓↓ resistance decreased; ↑↑ resistance increased

[2)] NA: Not applicable since growth is vigorous on copper even without NAC

Unless listed in Table B, the strains are the EUROSCARF deletion strains in the BY4741 background, having indicated genes replaced by a kanMX4 cassette.

References of Supporting Information

1. Toyn JH, Gunyuzlu PL, White WH, Thompson LA, Hollis GF (2000) A counterselection for the tryptophan pathway in yeast: 5-fluoroanthranilic acid resistance. Yeast 16: 553-560.
2. Vazquez HM, Vionnet C, Roubaty C, Conzelmann A (2014) Cdc1 removes the ethanolaminephosphate of the first mannose of GPI anchors and thereby facilitates the integration of GPI proteins into the yeast cell wall. Mol Biol Cell
3. Guillas I, Pfefferli M, Conzelmann A (2000) Analysis of ceramides present in glycosylphosphatidylinositol anchored proteins of Saccharomyces cerevisiae. Methods Enzymol 312: 506-515.
4. Friedlander R, Jarosch E, Urban J, Volkwein C, Sommer T (2000) A regulatory link between ER-associated protein degradation and the unfolded-protein response. Nat Cell Biol 2: 379-384.
5. Guillas I, Kirchman PA, Chuard R, Pfefferli M, Jiang JC, Jazwinski SM et al. (2001) C26-CoA-dependent ceramide synthesis of Saccharomyces cerevisiae is operated by Lag1p and Lac1p. EMBO J 20: 2655-2665.
6. Vionnet C, Roubaty C, Ejsing CS, Knudsen J, Conzelmann A (2011) Yeast cells lacking all known ceramide synthases continue to make complex sphingolipids and to incorporate ceramides into glycosylphosphatidylinositol (GPI) anchors. J Biol Chem 286: 6769-6779.
7. Westermann B, Neupert W (2000) Mitochondria-targeted green fluorescent proteins: convenient tools for the study of organelle biogenesis in Saccharomyces cerevisiae. Yeast 16: 1421-1427.
8. Lewis MJ, Nichols BJ, Prescianotto-Baschong C, Riezman H, Pelham HR (2000) Specific retrieval of the exocytic SNARE Snc1p from early yeast endosomes. Mol Biol Cell 11: 23-38.
9. Reggiori F, Pelham HR (2001) Sorting of proteins into multivesicular bodies: ubiquitin-dependent and -independent targeting. EMBO J 20: 5176-5186.

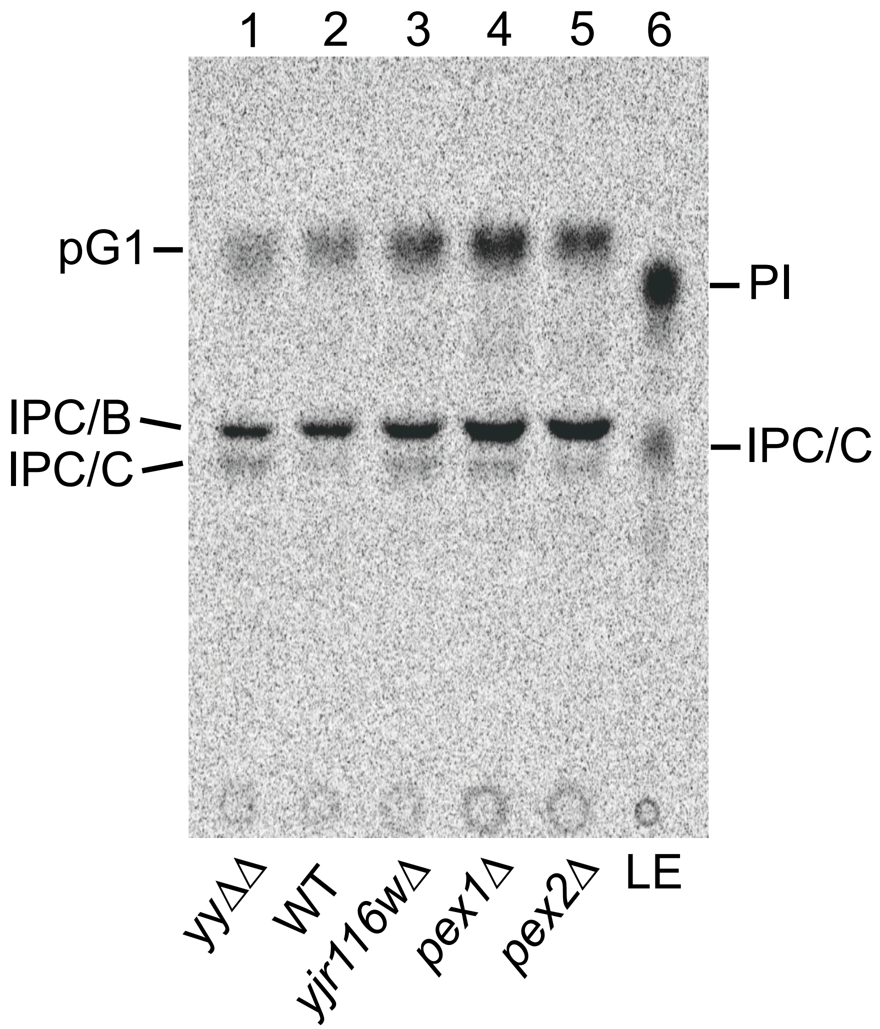

Fig A. GPI remodeling introduces normal amounts of ceramides into GPI anchors of *yjr116wΔ* and yyΔΔ cells. 10 OD_{600} units of cells were labeled with [^{3}H]inositol, GPI anchor lipids were isolated, analyzed by TLC and visualized using the phosphorimager. In addition also two *pex* mutants were tested. pG1 is PI with a C26:0 fatty acid in sn-2 position of glycerol. LE = lipid extract from WT cells containing PIs typically having C18:1 in sn-2.

Fig A

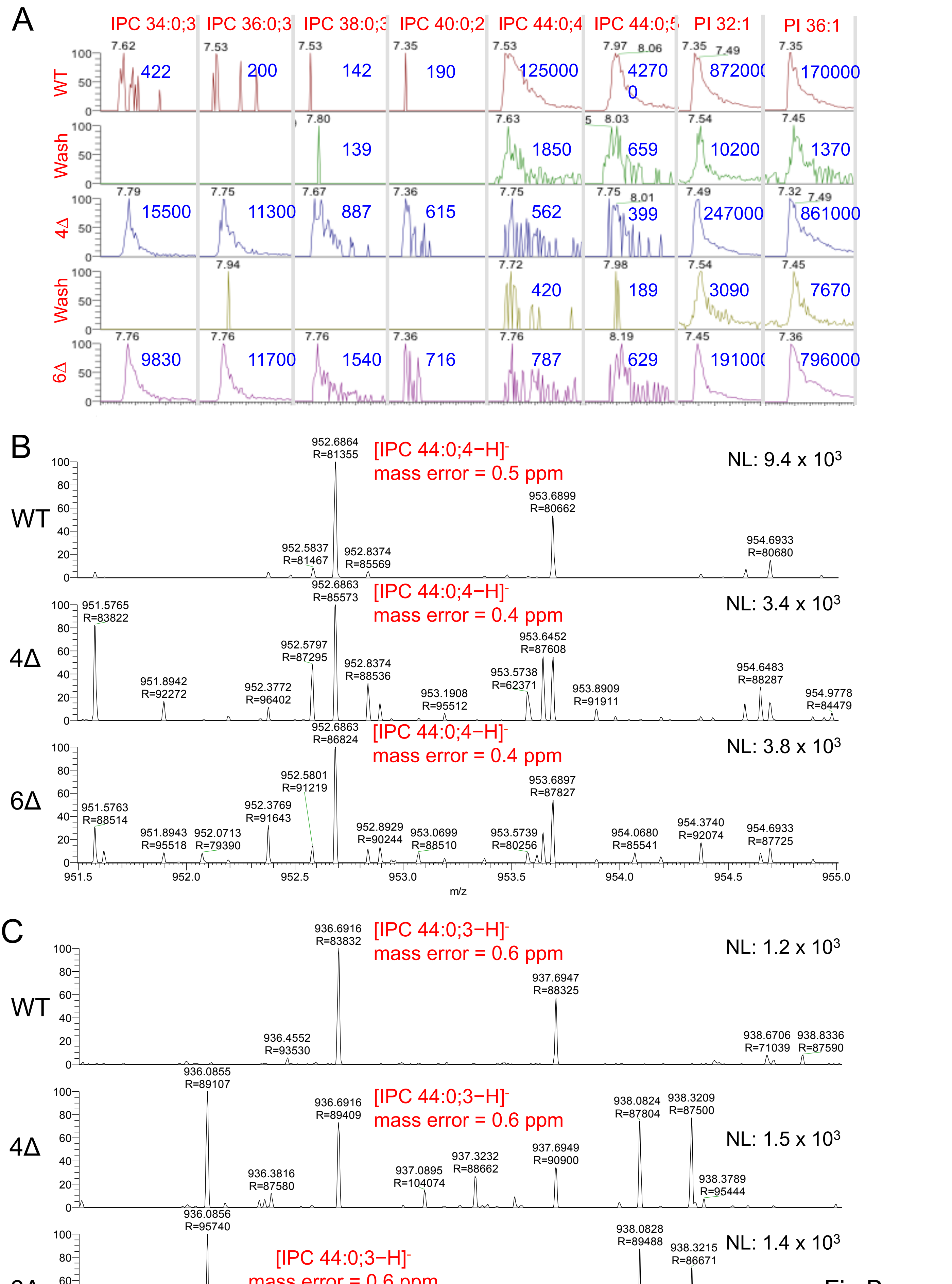

Fig B, panels A-C

D

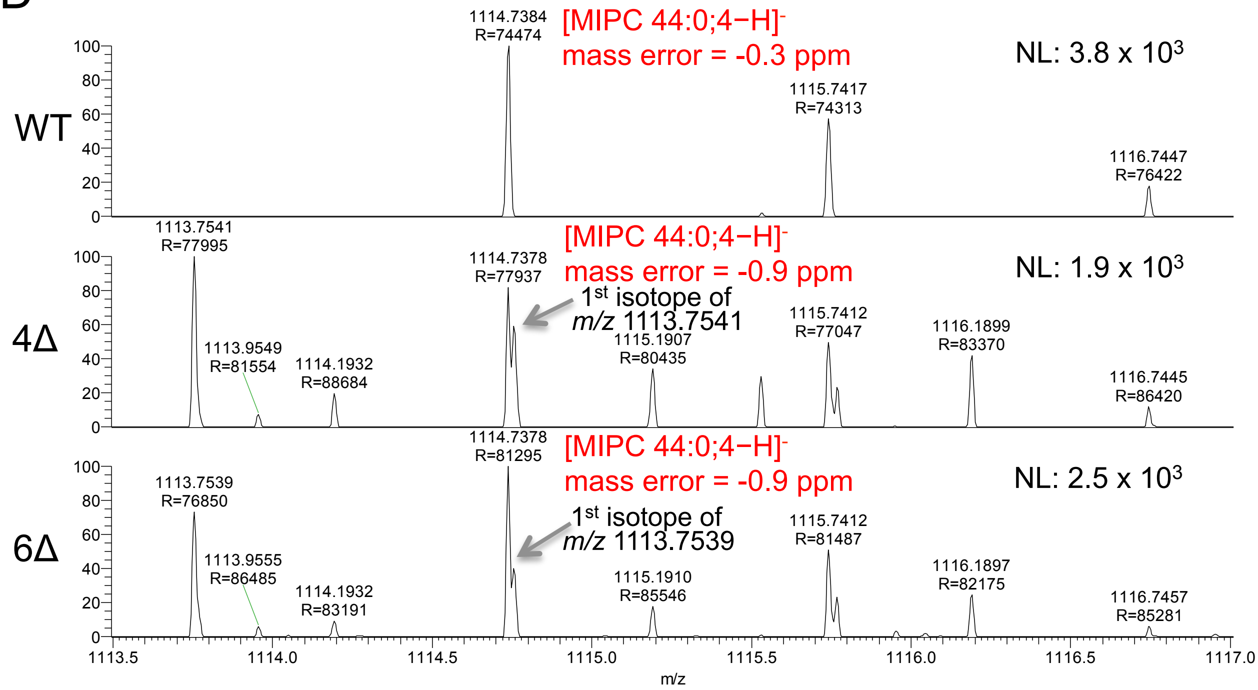

Fig B. A. Extracted ion chromatograms (XIC) of IPCs and PIs in the same NPLC-nanoESI-FTMS experiment as shown in Figs 2A, 2B, and 2E analyzed in the negative ion mode. Elution times of peak signals (in minutes) are in grey, apexes were set to 100 % and the intensity at the apex indicated in blue. B-D. In an independent experiment, lipid extracts from exponentially growing WT (W303), 4Δ and 6Δ cells were deacylated by mild NaOH treatment, desalted and analyzed by direct infusion MS. FTMS profiles for major IPCs and MIPC are shown. Fragmentation of $[IPC44:0;4\text{-}H]^-$ of 4Δ strain yielded the characteristic inositol-phosphate and mannosyl-inositol-phosphate fragments (not shown).

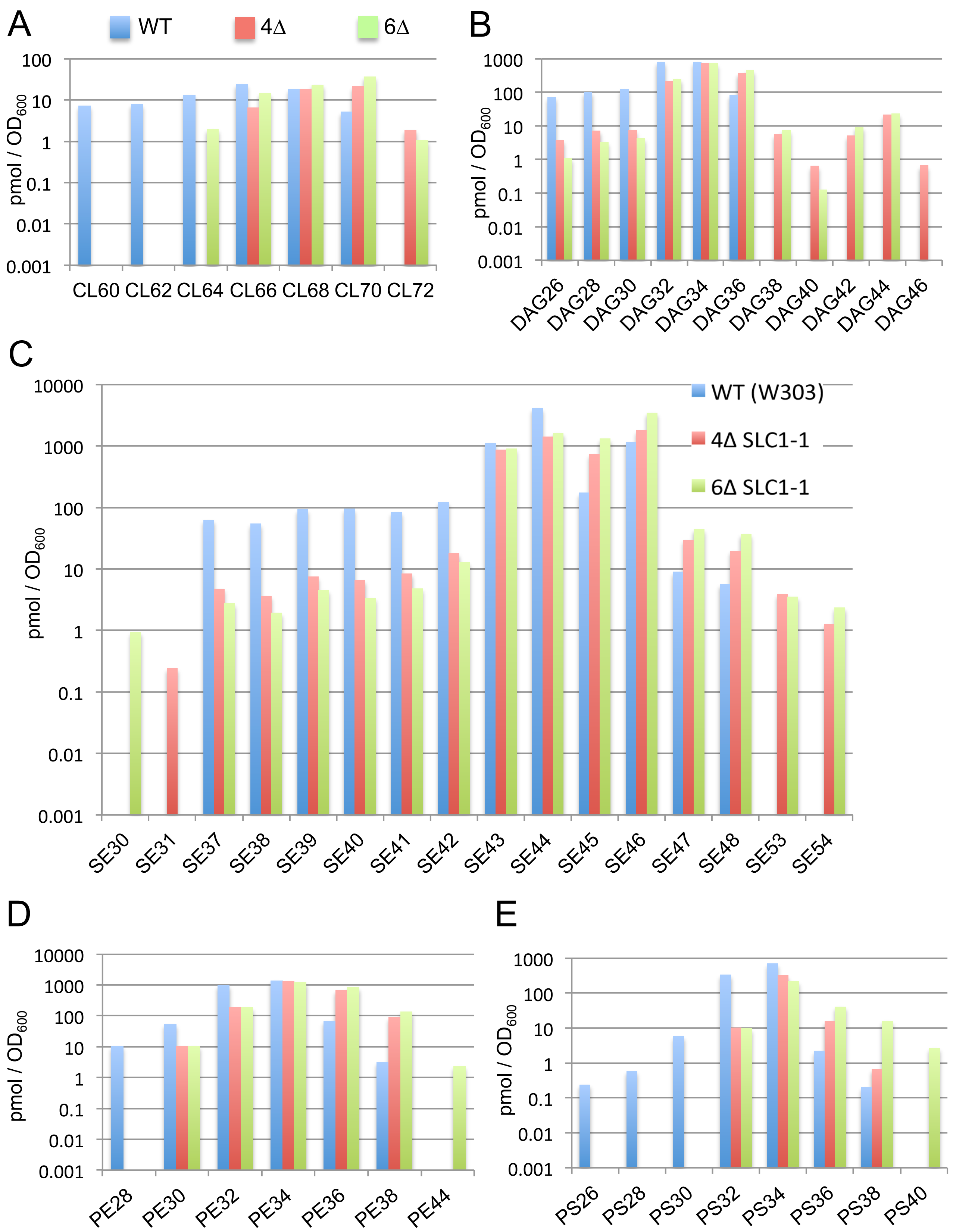

Fig C. Lipid profiles of W303-1A, 4Δ and 6Δ cells were obtained by direct infusion mass spectrometry using the negative ion mode. Quantifications of lipids were done by comparison of intensities with the signals of internal standards belonging to the same lipid class. A. Cardiolipin (CL); B. Diacylglycerol (DAG); C. Steryl ester (SE); D. Phosphatidylethanolamine (PE); E. Phosphatidylserine (PS).

Fig C

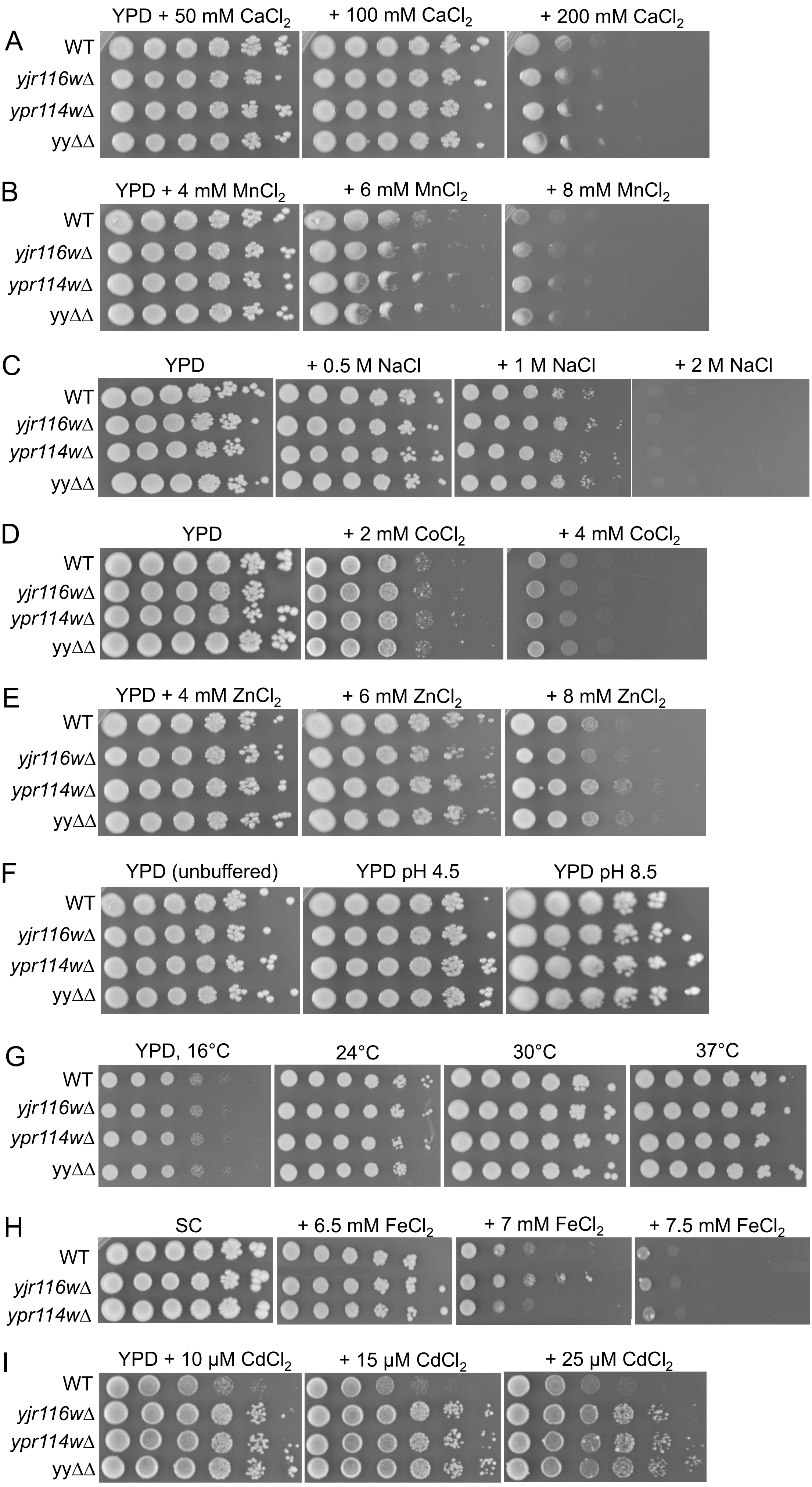

A
YPD + 50 mM $CaCl_2$
+ 100 mM $CaCl_2$
+ 200 mM $CaCl_2$
WT
*yjr116w*Δ
*ypr114w*Δ
yyΔΔ
B
YPD + 4 mM $MnCl_2$
+ 6 mM $MnCl_2$
+ 8 mM $MnCl_2$
C
YPD
+ 0.5 M NaCl
+ 1 M NaCl
+ 2 M NaCl
D
YPD
+ 2 mM $CoCl_2$
+ 4 mM $CoCl_2$
E
YPD + 4 mM $ZnCl_2$
+ 6 mM $ZnCl_2$
+ 8 mM $ZnCl_2$
F
YPD (unbuffered)
YPD pH 4.5
YPD pH 8.5
G
YPD, 16°C
24°C
30°C
37°C
H
SC
+ 6.5 mM $FeCl_2$
+ 7 mM $FeCl_2$
+ 7.5 mM $FeCl_2$
I
YPD + 10 μM $CdCl_2$
+ 15 μM $CdCl_2$
+ 25 μM $CdCl_2$

Fig D

Fig D. Growth of WT and mutants on SC media in presence of different metal ions, salt concentrations, H^+ concentrations and temperatures. A. $CaCl_2$; B. $MnCl_2$; C. NaCl; D. $CoCl_2$; E. $ZnCl_2$; F. Variable pH; G. Variable temperature; H. $FeCl_2$; I. $CdCl_2$. (H – S288c background (RH3435, FBY7487, FBY7488), all other panels use strains with BY4742 background).

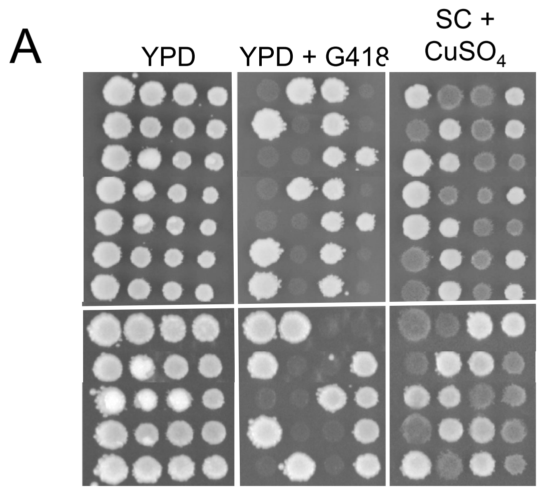

Fig E. A. *Yjr116wΔ* cells were crossed with BY4741 WT cells, tetrads were dissected and replicated onto YPD, YPD plus G418 or YPD plus 3 mM Cu^{2+}. B - E. Serial dilutions of WT and mutant cells on SC plates containing indicated concentrations of $CuSO_4$, NAC and N-acetyl-serine (NAS). Based on the rescue by NAC and the susceptibility to copper, the strains in panel E were categorized in 5 classes as shown in Table D.

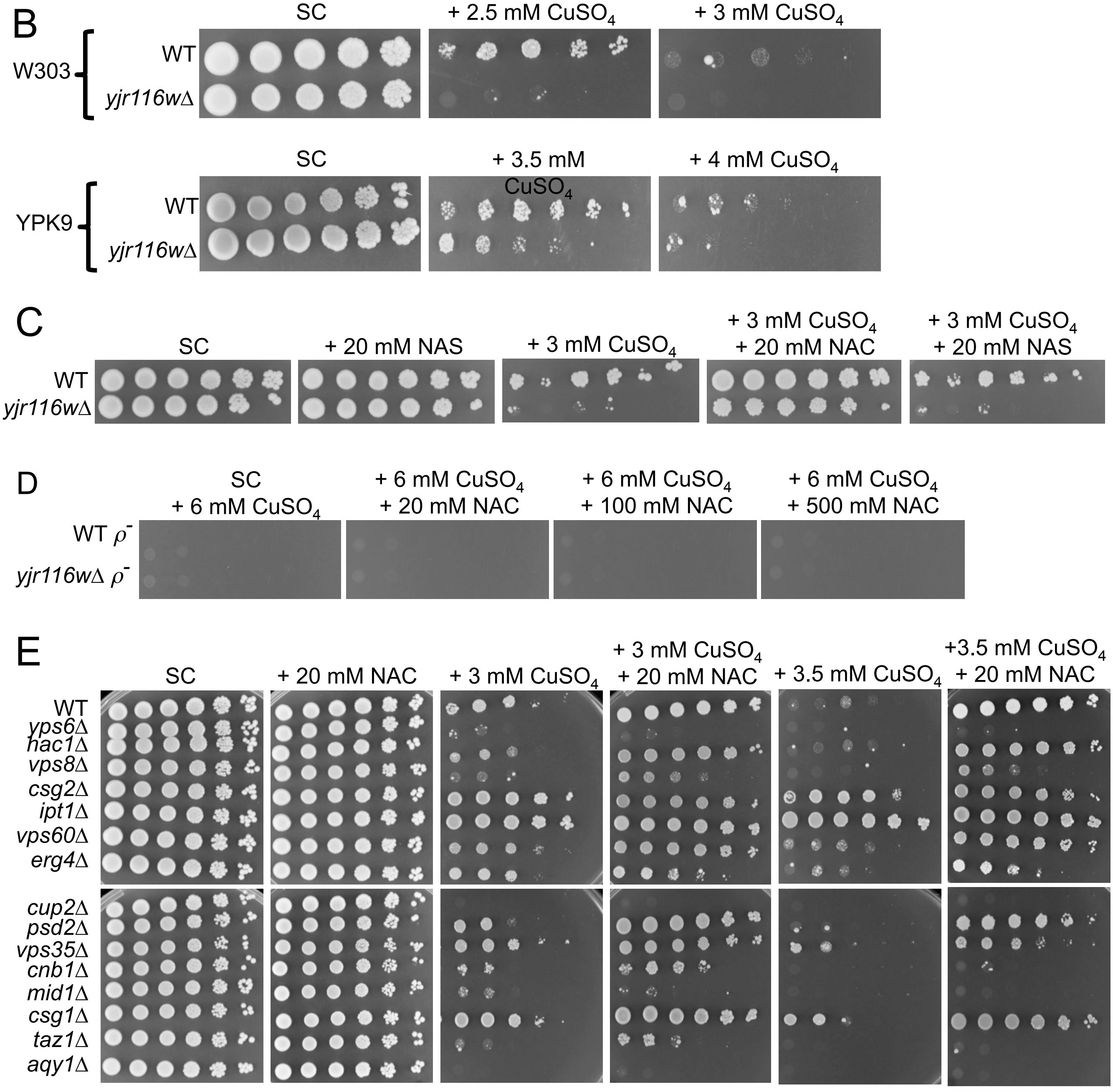

Fig E

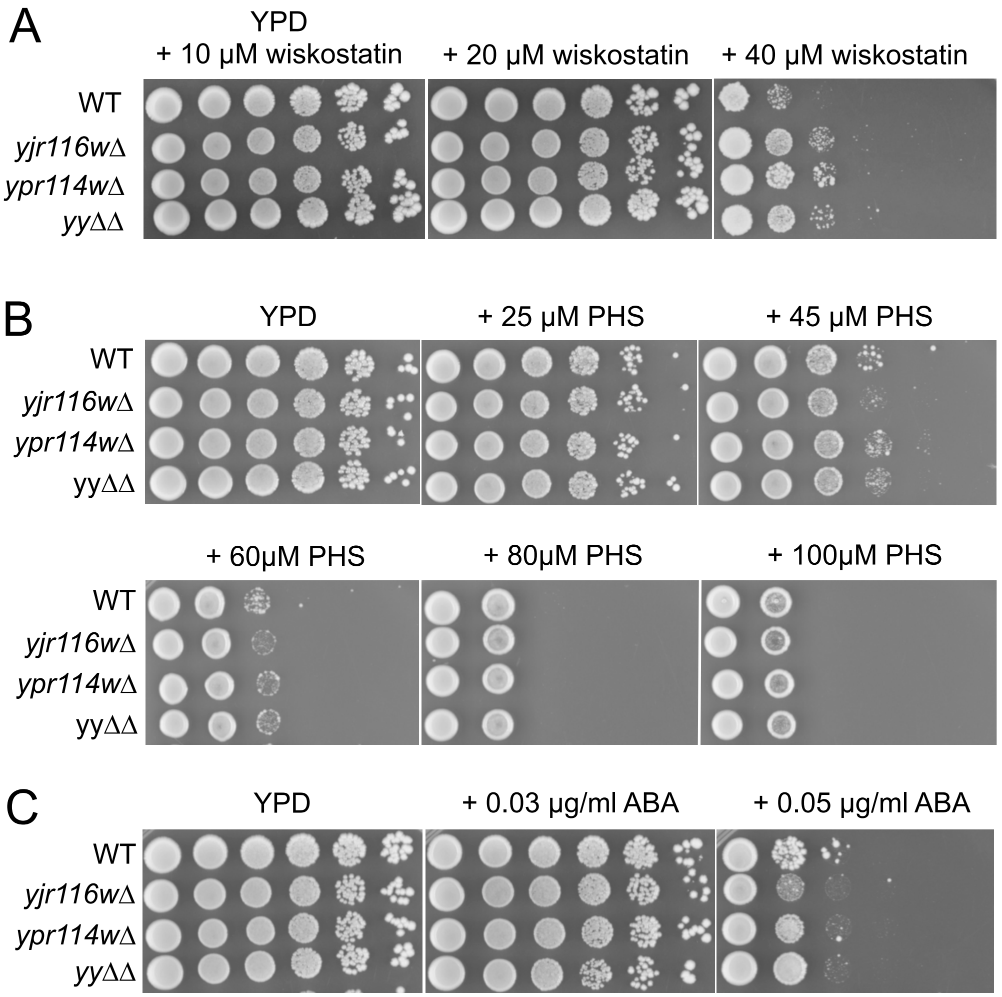

Fig F. Growth of WT and mutants on different concentrations of Wiskostatin (A), PHS (B), and Aureobasidin A (AbA)(C).

Fig F

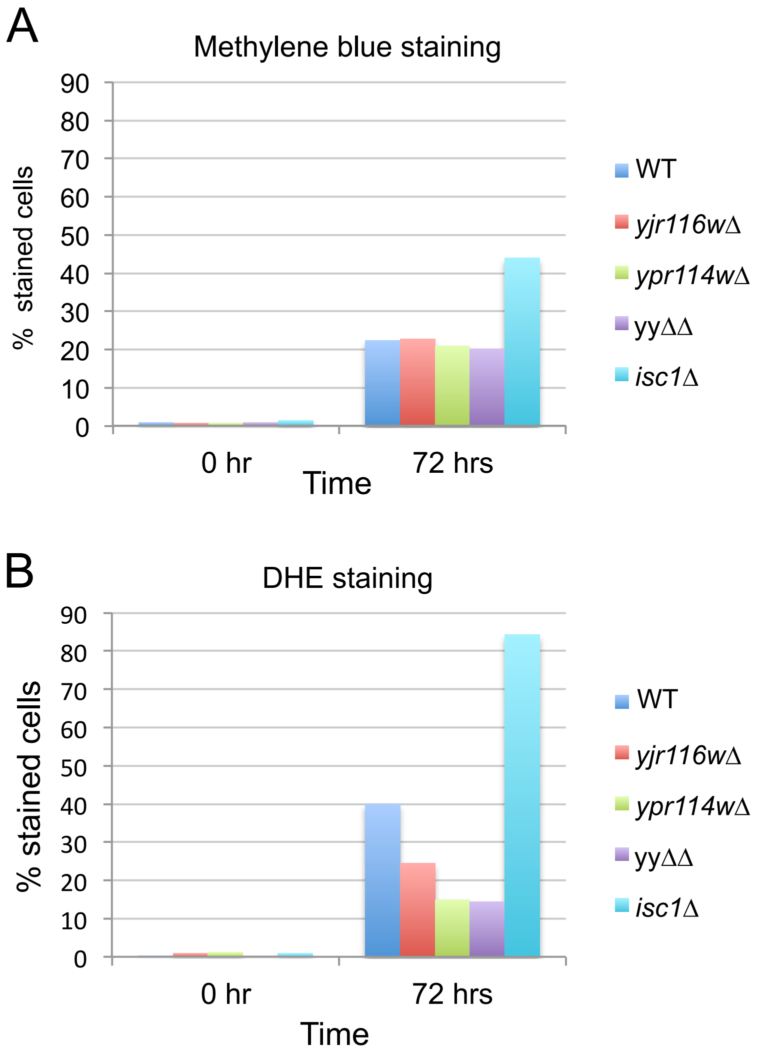

Fig G. Determination of percentages of dead and of superoxide anion accumulating cells before and after 72 h of chronological aging in water. A. Cells grown to exponential phase in YPD were placed for 0 or 72 h into water and were then stained with methylene blue during 2 minutes. Methylene blue stained cells (>200 cells/strain) were counted under microscope within 5 min. B. Same as A but cells were stained with DHE.

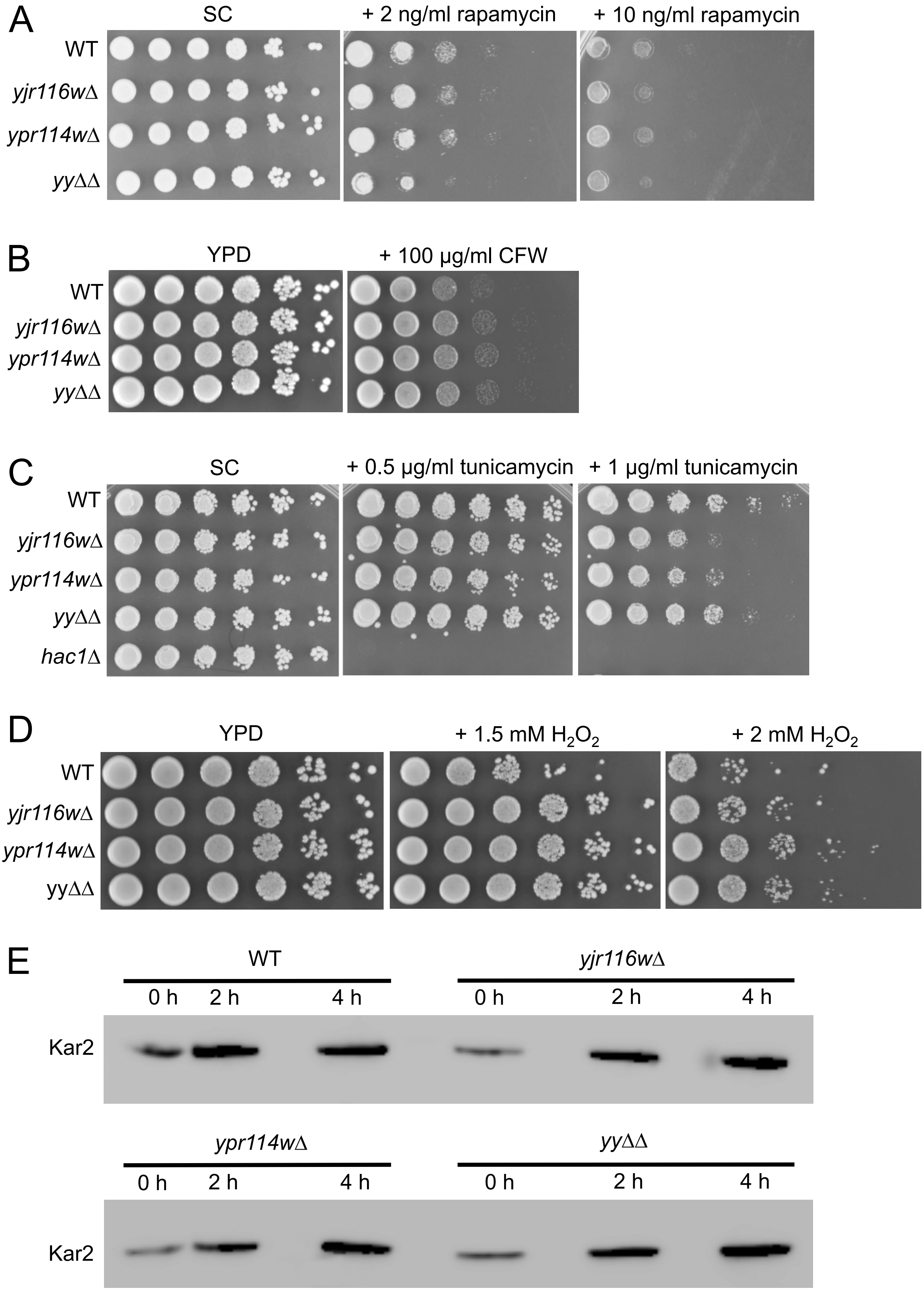

Fig H. Stress resistance of *yjr116wΔ* and *ypr114wΔ* mutants. Serial dilutions of exponentially growing cells were plated on YPD with rapamycin (A), CFW (B), tunicamycin (C) and H_2O_2 (D). E. Western blot analysis of Kar2 protein after exposing cells for 0 - 4 h to tunicamycin (1 µg ml^{-1}).

Fig H

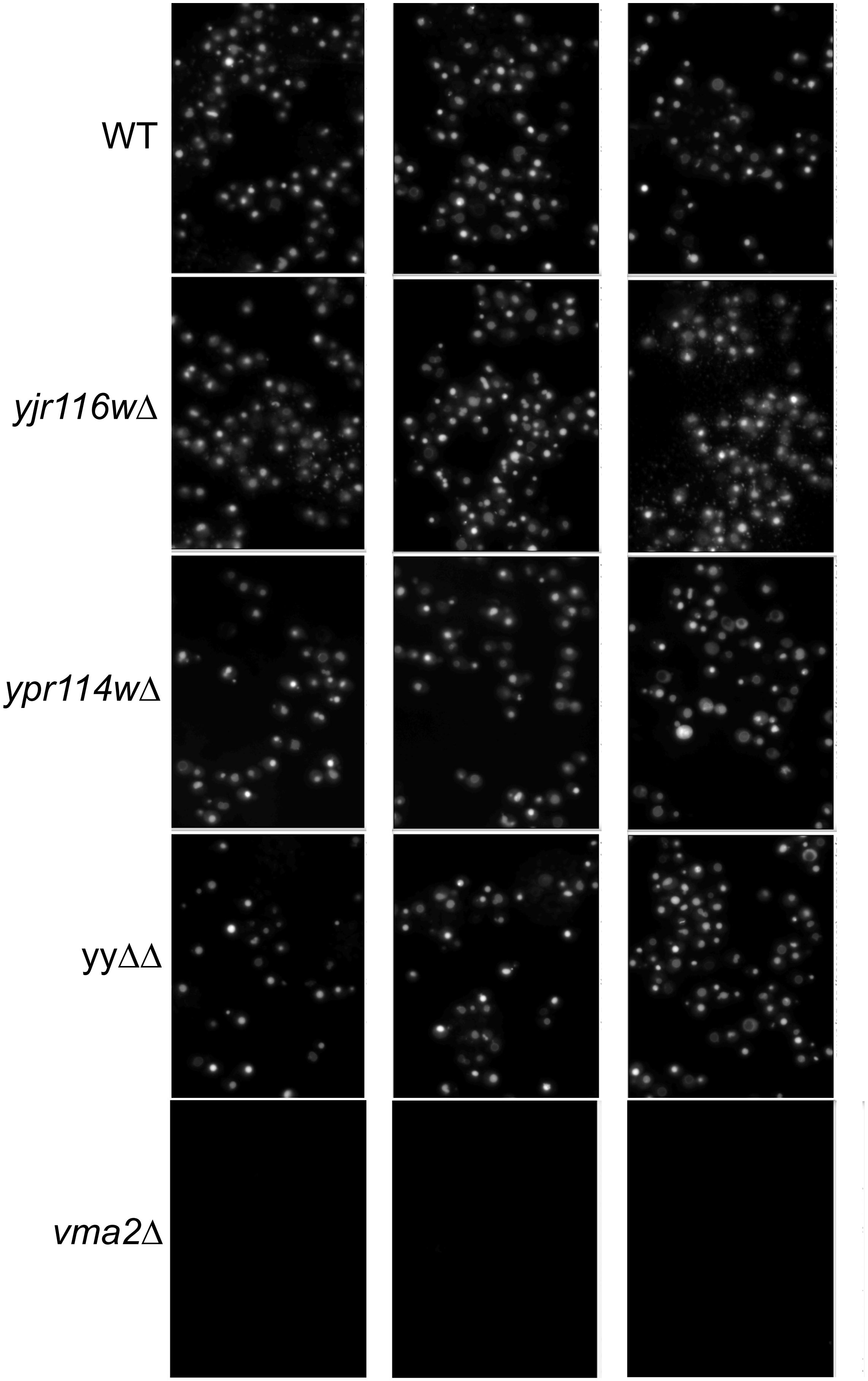

Fig I. WT and mutant cells grown in YPD were stained with quinacrine as described in Experimental Procedures in order to assess their capacity to acidify the vacuole.

Fig I

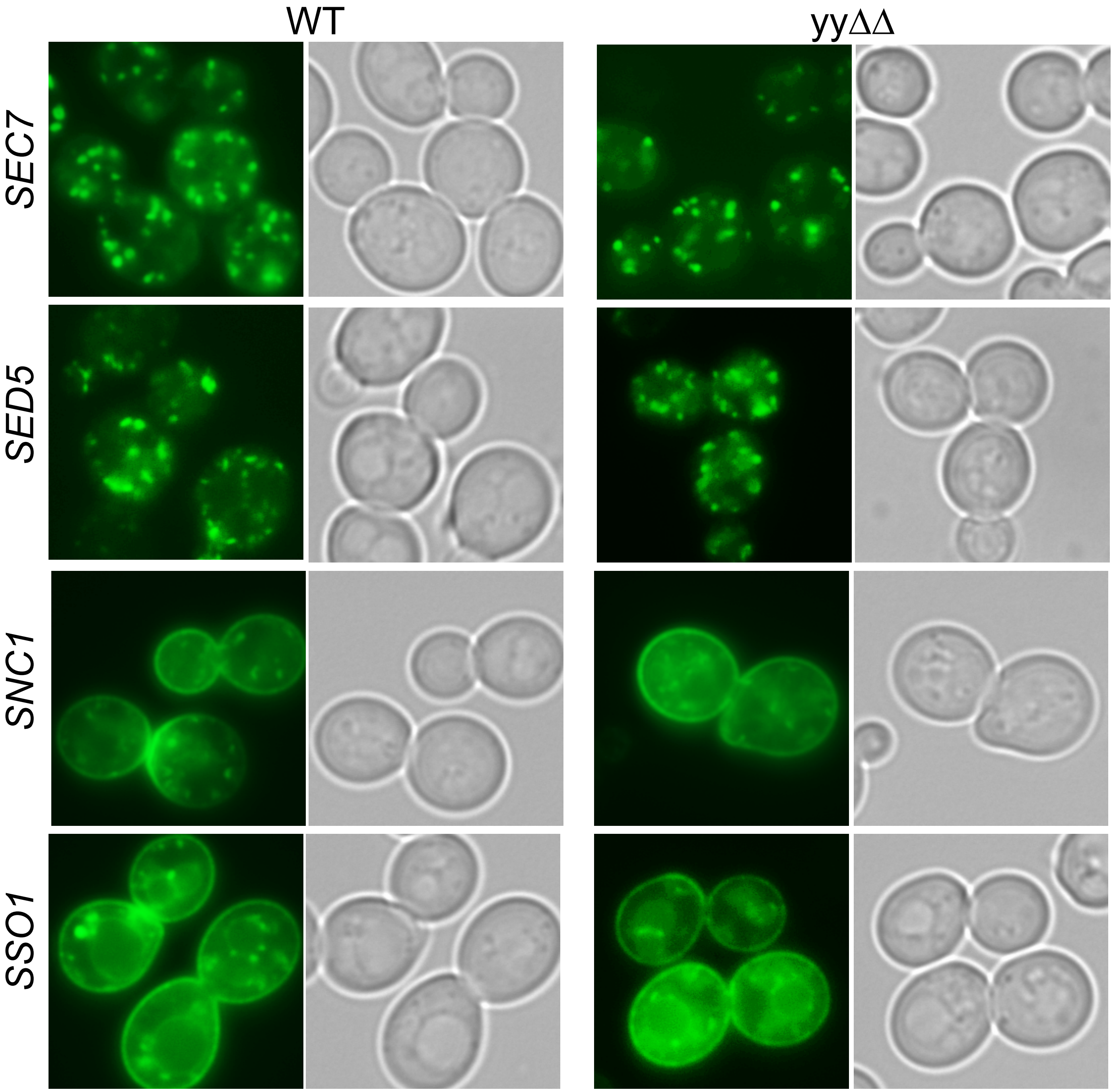

Fig J. Morphology of organelles of the secretory pathway. WT and yyΔΔ cells expressing SEC7-GFP and GFP-SED5, staining the Golgi, GFP-SNC1 and GFP-SSO1, SNARE proteins cycling between Golgi, endosomes and plasma membrane were viewed by fluorescence microscopy. All GFP constructs were expressed from centromeric plasmids under either the *ADH1* or *TPI1* promoter.

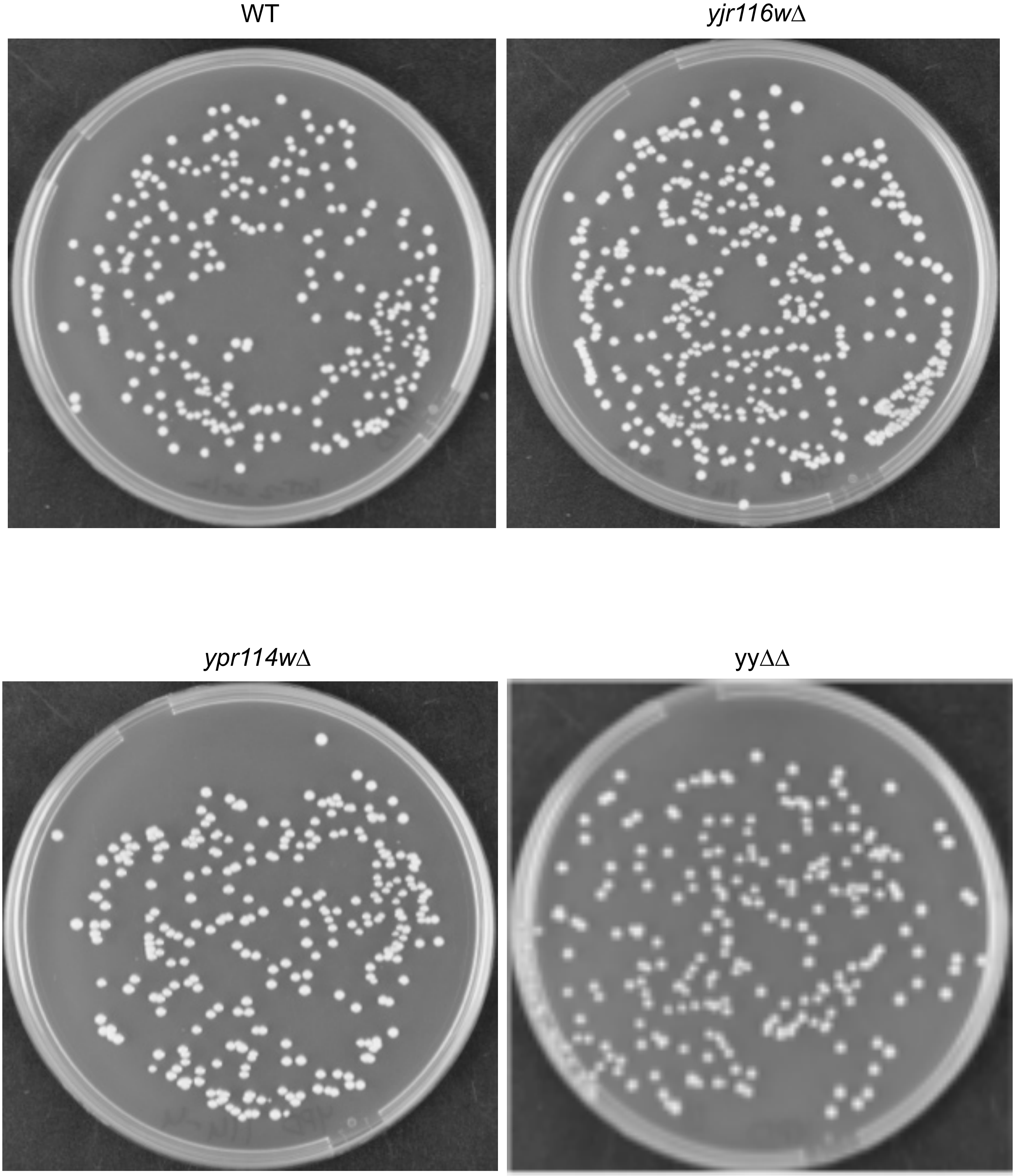

Fig K. Cells were plated on YPD to evaluate whether they had spontaneously been overgrown by petite strains.

Fig K
